# Applications of multi‐omics analysis in human diseases

**DOI:** 10.1002/mco2.315

**Published:** 2023-07-31

**Authors:** Chongyang Chen, Jing Wang, Donghui Pan, Xinyu Wang, Yuping Xu, Junjie Yan, Lizhen Wang, Xifei Yang, Min Yang, Gong‐Ping Liu

**Affiliations:** ^1^ Key Laboratory of Nuclear Medicine Ministry of Health Jiangsu Key Laboratory of Molecular Nuclear Medicine Jiangsu Institute of Nuclear Medicine Wuxi China; ^2^ Co‐innovation Center of Neurodegeneration Nantong University Nantong China; ^3^ Shenzhen Key Laboratory of Modern Toxicology Shenzhen Medical Key Discipline of Health Toxicology (2020–2024) Shenzhen Center for Disease Control and Prevention Shenzhen China; ^4^ Department of Pathophysiology School of Basic Medicine Key Laboratory of Ministry of Education of China and Hubei Province for Neurological Disorders Tongji Medical College Huazhong University of Science and Technology Wuhan China

**Keywords:** biomarker, machine learning and deep learning, multi‐omics, neurodegenerative disease, precision medicine

## Abstract

Multi‐omics usually refers to the crossover application of multiple high‐throughput screening technologies represented by genomics, transcriptomics, single‐cell transcriptomics, proteomics and metabolomics, spatial transcriptomics, and so on, which play a great role in promoting the study of human diseases. Most of the current reviews focus on describing the development of multi‐omics technologies, data integration, and application to a particular disease; however, few of them provide a comprehensive and systematic introduction of multi‐omics. This review outlines the existing technical categories of multi‐omics, cautions for experimental design, focuses on the integrated analysis methods of multi‐omics, especially the approach of machine learning and deep learning in multi‐omics data integration and the corresponding tools, and the application of multi‐omics in medical researches (e.g., cancer, neurodegenerative diseases, aging, and drug target discovery) as well as the corresponding open‐source analysis tools and databases, and finally, discusses the challenges and future directions of multi‐omics integration and application in precision medicine. With the development of high‐throughput technologies and data integration algorithms, as important directions of multi‐omics for future disease research, single‐cell multi‐omics and spatial multi‐omics also provided a detailed introduction. This review will provide important guidance for researchers, especially who are just entering into multi‐omics medical research.

## Graphical abstract

Multi‐omics contains genomics, transcriptomics, proteomics, metabolomics, etc. And the experimental design considers the disease characteristics, disease model, sample size and phenotypic data. After the data integration by the approach of correlation, network and machining learning, the multi‐omics can be applied in diagnosis, biomarkers, targets of human disease and target discovery of natural compound.

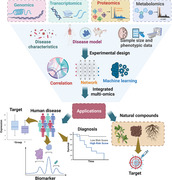

## 1 INTRODUCTION

As medical technology rapidly evolves, researchers need to conduct an in‐depth analysis of the pathogenesis of diseases. The omics technologies provide a high‐throughput screening method to uncover the detailed biological information of human diseases efficiently and rapidly. Usually, the omics technologies included genomics, transcriptomics, proteomics, metabolomics, single‐cell transcriptomics, single‐cell multi‐omics, spatial transcriptomics, and others. Each type of omics data provides differentially expressed disease‐associated molecules that can serve as biomarkers during disease progression and provide insight into which biological pathways or processes differ between disease and control groups. However, it should be noted that single omics technology cannot give full play to its application value in disease research. Mutations that occur in DNA will affect the expression of proteins, which may result in partial or total loss of some functions, leading to biological defects. But it is hard to tell the extent of the loss of function based on the genome alone. The level of gene expression, and ultimately how much protein is produced, both are related to the state of the disease. In addition, the occurrence of a disease may be related to a mutation in a gene, or an error in the transcription, translation, or other processes of the gene. In real research, one type of omics research can only carry out the correlation analysis with diseases, mainly reflecting the change of disease process and cannot explain the causal relationship. In Alzheimer's disease, for example, even if a biochemical molecule is statistically associated with the disease, it does not explain the complex mechanisms underlying the disease.

Integration of different types of omics data can elucidate underlying pathogenic changes of the disease, which can then be verified in further molecular researches. By integrating multi‐omics, scientists can filter out novel associations between biomolecules and disease phenotypes, identify relevant signaling pathways, and establish detailed biomarkers of disease. Therefore, the integration of various omics data will facilitate the match of associations between molecular‐disease and phenotype‐environmental factors. For instance, the molecular profiling in primary tissues through multi‐omics integration to reveal molecular mechanisms of the progress of disease,^1^ estimation of the biological age of organs (liver, kidneys, etc.), and systems (immune and metabolic systems) by a multi‐omics approach for the assessment of aging status.^2^ Moreover, since the occurrence and development of diseases are not only affected by the latest gene mutations, but also by the environmental factor, genetic background, gene regulation, and so on, the research of multi‐omics from different levels has also laid the foundation for the development of systems biology technology, such as multi‐omics reveals systems biology in cardiovascular disease.^3^ Recently, single‐cell omics and spatial omics provide more detail information about the mechanisms of interactions between intracellular and intercellular molecules that control development, physiology, and pathology.^4^ The integration analysis of single‐cell transcriptomics and spatial transcriptomics has successfully resolved the logic underlying spatially organized immune‐malignant cell networks in human colorectal cancer.^5^ However, although many high‐throughput omics technologies have made good research progress in the medical field, there are few systematic introductions on how multi‐omics should be carried out in disease research, such as experimental design, data integration, analysis tools, and so on,^6^ especially for researchers who are just coming or preparing to conduct multi‐omics research.

In this review, we briefly described the types of multi‐omics used in human disease research, and focused on the experimental design, data integration, application, challenges, and future development directions of multi‐omics. In particular, we provide a detailed description of the experimental design and data integration in multi‐omics research. We also have offered open‐source tools that can be used to analyze and integrate multi‐omics data, which will greatly benefit researchers who are unfamiliar with algorithms. This review will be described in sequence from omics classification, experimental design, data integration, disease application, challenges and future development directions, and conclusion, which will provide valuable guidance for researchers or newcomers in the field of multi‐omics.

## 2 THE CATEGORIES OF OMICS

**FIGURE 1:**
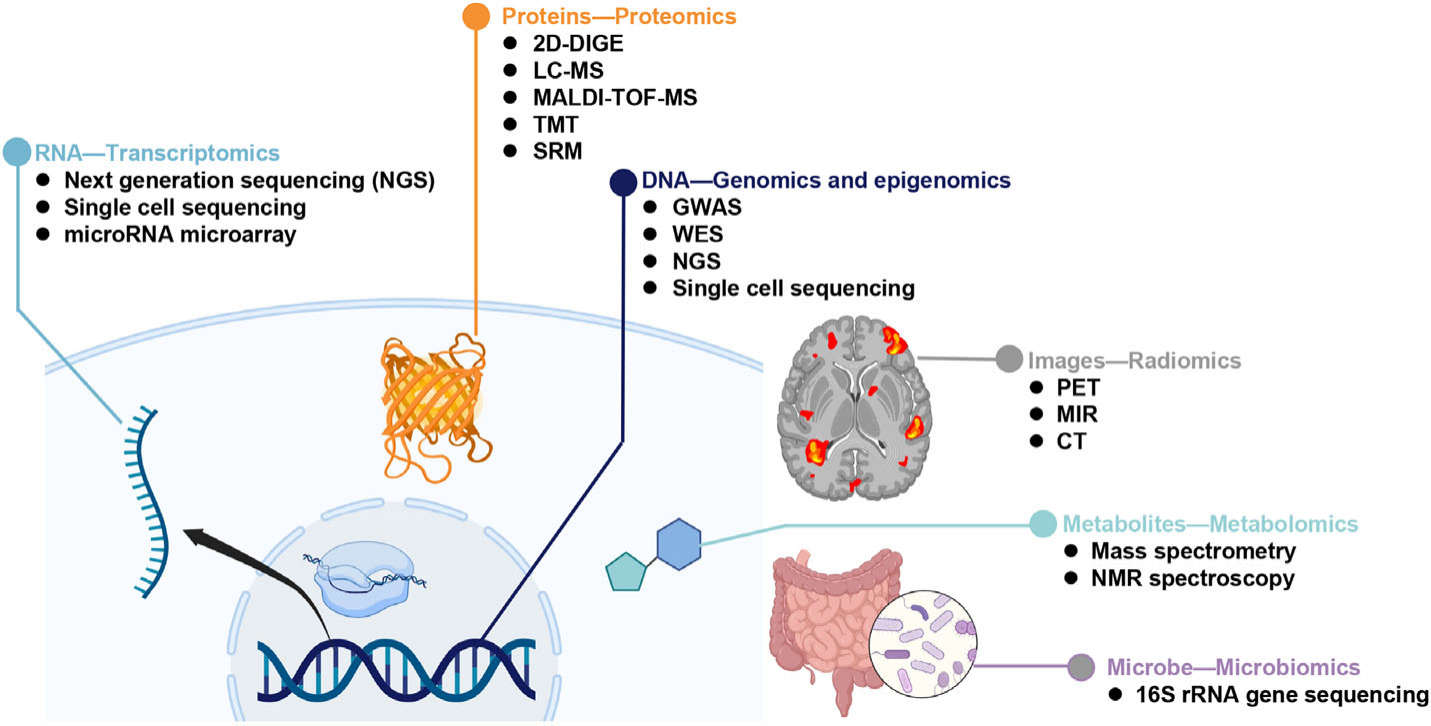
Multi‐omics approaches in disease research. Here lists the currently main available methods for each omics. RNA‐transcriptomics including next‐generation sequencing (NGS), single‐cell sequencing, and microRNA microarray. Proteins‐proteomics including 2D differential gel electrophoresis (2D‐DIGE), liquid chromatography‐mass spectrometry (LC‐MS), matrix‐assisted laser desorption ionization time‐of‐F (MALDI‐TOF‐MS), tandem mass tag (TMT), and selected reaction monitoring (SRM). DNA‐genomics and epigenomics including genome‐wide association studies (GWAS), whole‐exome sequencing (WES), next‐generation sequencing (NGS), and single‐cell sequencing. Images—radiomics including positron emission tomography (PET), magnetic resonance imaging (MRI), and computed tomography (CT). Metabolites—metabolomics including mass spectrometry and NMR spectroscopy. Microbe—microbiomics including 16S rRNA gene sequencing.

With the development of high‐throughput technology, there are now many types of omics in the medical field, mainly including transcriptomics, proteomics, genomics and epigenomics, single‐cell omics, spatial transcriptomics, radiomics, metabolomics, and microbiomics, whose applications mainly involve cell molecular level, intestinal microbial system, and pathological imaging, and so on (Figure 1).

### 2.1 Genomics

Genomics is the application of omics in entire genomes, which aims to collect character and quantify all genes of an organism, uncover their interrelationship, and influence on the organism. Genomics is the earliest and most common application in medicine, such as The Human Genome Project.^7^ Genomics usually contains the components of high‐throughput DNA sequencing, sequence assembly, and genome annotation.^8^ Genome‐wide association study (GWAS) is a typical application of genomics to find out the existing sequence variation in the whole human genome, namely, single nucleotide polymorphism (SNP), and screen out disease‐related SNPs (https://www.ebi.ac.uk/gwas/). The associated technologies contained genotyping array,^9^ third‐generation sequencing for whole‐genome sequencing,^10^ and exome sequencing.^11^ In GWAS studies, the millions of genetic variants across the genomes of multiple individuals are tested to identify genotype−phenotype associations.^12^ Despite GWAS can identify novel disease‐associated susceptibility genes, biological pathways and translate these findings into clinical care, most of the acquired variants and genes have no direct biological relevance to disease.^13^

### 2.2 Transcriptomics

Transcriptomics refers to the study of the expression of all the RNAs from a given cell population, which offer a global perspective on molecular dynamic changes induced by environment factors or pathogenic agents. The transcriptome of RNAs detection includes protein‐coding RNAs (mRNAs), long noncoding RNAs, short noncoding RNAs (microRNAs, small‐interfering RNAs, small nuclear RNAs, piwi‐interacting RNAs, and enhancer RNAs), and circular RNAs. In addition to mRNAs, noncoding RNAs also have associations with diseases,^14^ such as diabetes,^15^ cancer,^16^ and so on, circular RNAs have a link with cardiovascular disease,^17^ CNS disease,^18^ and cancer.^19^ Since mRNA accounts for 1%−4% of the overall transcript, the use of the transcriptome to study the impact of noncoding RNAs on disease is an important trend in the future. The usually used technology for transcriptomics is RNA‐seq, which can quality and quantify RNA transcripts from little RNA sample.^20^ Currently, with the development of technology, the single‐cell transcriptome (single‐cell RNA sequencing [scRNA‐seq]) becomes hot, which can detect the transcripts of specific cell types in diseases (such as cancer,^21^ Alzheimer's disease,^22^ etc.).

### 2.3 Proteomics

Proteomics enables the maximum identification and quantification of all proteins in cells or tissues. Because the level of gene transcription is often affected by post‐transcriptional modifications, the RNA analysis usually lacks correlation with protein expression.^23^ Thus, proteomics can quantify the protein expression and provide the directly associated information with the environment change or disease progression. The large‐scale study of proteins can be detected by mass spectrometry‐based method, affinity proteomics, protein chips, and reverse‐phased protein microarrays.^24^ The mass‐based proteomics is widely used in modern medical research and classified as stable isotope labeling proteomics and label‐free proteomics, while labeled proteomics can shorten the detection time of mass spectrometry significantly and reduce the batch effect between samples.^25^ In addition, the protein and protein interaction can also be identified by a combination of immunoprecipitation and mass spectrometry, for example, purification of target proteins by antibodies (BMPR‐1B), and followed by the detection of interacting unknown proteins by mass spectrometry, finally, acquired the interacted proteins (GDF5, GDF9, RhoD, and HSP10).^26^ Noteworthy, the post‐translational modifications can be broadly found in proteins after translation, such as phosphorylation, glycosylation, ubiquitination, acetylation, and nitrosylation,^27^ and those modifications are critical to intracellular signal transduction, protein transport, and enzyme activity.^28^ Studying a set of modified proteins can better understand the progression of the disease, such as phosphoproteomics uncover the novel mechanism in type 2 diabetes,^29^ lung adenocarcinoma,^30^ nonalcoholic steatohepatitis,^31^ Alzheimer's disease,^32^ and so on.

### 2.4 Metabolomics

Metabolomics (usually containing untargeted metabolomics and targeted metabolomics) focuses on the study of a set of small molecule metabolites derived from cellular biological metabolic processes. Those metabolites include small molecule substrates, intermediates, and end products of cellular metabolism, such as carbohydrates, fatty acids, and amino acids. In general, metabolite analysis can immediately reflect dynamic changes in cell physiology, and abnormal metabolite level or ratio may induce disease. Metabolomics will help to elucidate the mechanisms of disease progression.^33^, ^34^, ^35^ Additionally, there has a quantifiable correlation between metabolomics and other omics (genomics, transcriptomics, proteomics, etc.), such as mRNA count can predict metabolite level,^36^ gut bacteria has a correlation with amino acids level in patients with fibromyalgia,^37^ the expressions of creatine kinase and mitochondrial protein are consistent with acylcarnitine and acetyl‐CoA in human hypertrophic cardiomyopathy.^38^ Moreover, the Human Metabolome Database (https://hmdb.ca/) is a free database containing detailed information of small molecule metabolites in the human body that can be used for metabolomics research,^39^ and the corresponding metabolites analysis can be performed by MetaboAnalyst 5.0 (https://www.metaboanalyst.ca/, a free platform for metabolomics analysis).^40^ Finally, the associated databases and related technologies for metabolomics include nuclear magnetic resonance (NMR)^41^ and mass spectrometry (MS)‐based methods (gas chromatograph–mass spectrometer (GC‐MS), liquid chromatography tandem mass spectrometry(LC‐MS), and capillary electrophoresis–mass spectrometry (CE‐MS)).^42^, ^43^

### 2.5 Single‐cell omics

Recently, the rapid development of single‐cell sequencing technology has become popular in medical research. Single‐cell sequencing has great power in elucidating the heterogeneity of transcriptomics, genomics, and epigenomics in cellular populations, and corresponding changes in those levels.^44^ To be specific, single‐cell transcriptomics aims to accurately understand the transcriptome status of heterogeneous cell populations. Single‐cell genomics reveals genetic heterogeneity from cells with or without mutation accumulation. Single‐cell epigenomics is used for detecting footprints of differentiation of individual cells. For instance, single‐cell transcriptome identified distinct transcriptional responses in lung cancer cell lines (CCLs), which were sensitive and insensitive to receptor tyrosine kinase inhibitors, and found distinct transcriptional modules that may be associated with early drug resistance.^45^ Single‐cell targeted DNA sequencing obtained the information that cells that acquired RAS/MAPK mutations have a resistance to FLT3 inhibitors in acute myeloid leukemia.^46^ Single‐cell ChIP‐sequencing revealed a distinct H3K27me3 pattern in resistant cells obtained from breast cancer.^47^ In addition, single‐cell proteomics, another research center stage after single‐cell sequencing,^48^ enables qualitative and quantitative analysis of protein composition within individual cells to reveal fundamental differences in the types and states of different individual cells, such as tumor heterogeneity, stem cell differentiation, germ cell development, and circulating tumor cells.

Moreover, single‐cell analysis has a tendency to enter the multi‐omics age. By integrating different types of molecular data (such as data on mutations, mRNAs, and proteins) from single‐cell, a comprehensive understanding of cellular processes can be achieved. Mahdessian et al. have systematically identified cell cycle‐associated heterogeneously expressed proteins at the mRNA and protein levels in single‐cell level.^49^ Meanwhile, there are many assay platforms for single‐cell analysis, such as 10×Chromium Single Cell Gene Expression Solution, BD Rhapsody™ Single‐Cell Analysis System, and so on.

### 2.6 Spatial transcriptomics

Spatial transcriptome (ST) is a technology that preserves the spatial location of tissues and simultaneously resolves transcriptome information in tissue sections. This technique is used to analyze and describe the expression profiles of specific cell types in a spatial dimension to understand transcription differences among organs, tissues, and pathological states. ST can also be able to parse transcripts of tissues at different spatial locations. For example, ST has successfully pinpointed the type of testicular cells in a single spermatogenic tubule in mammals.^50^ By detecting spatial gene expression in HER2‐positive breast tumors, ST spatially maps tumor‐associated cell types and obtains tertiary lymphoid‐like structures whose signal intensity correlates with overall survival.^51^ The ST technologies include next‐generation sequencing (NGS)‐based techniques, where positional information is encoded onto transcripts prior to NGS sequencing, and imaging‐based methods include in situ sequencing, where transcripts are amplified and sequenced in tissue, and in situ hybridization (ISH)‐based methods, where imaging probes are sequentially hybridized in tissue. Moreover, spatial omics techniques have evolved to the spatial multi‐omics stage. The combination of spatial transcriptomics and proteomics has identified the multicellular mechanisms and early neurodegenerative pathways in the pathogenesis of progressive multiple sclerosis.^52^

### 2.7 Epigenomics

Epigenomics focuses on the reversible modifications of DNA or histones that affect gene expression, and the main modification, including DNA methylation or histone modification.^53^ The modifications of DNA and histone act as important roles to regulate gene expression and cellular processes (such as development or differentiation).^54^ Those modifications usually can be influenced by environmental or genetic factors, and sometimes may be lasting or heritable.^55^ Many researchers reported that epigenetic modifications have an association with diseases, such as type 2 diabetes,^56^ cardiovascular disease,^57^ cancer,^58^ and so on. Epigenetic marks are often tissue‐specific, and the related research program contains Reference Epigenome Mapping Centers.^59^ NGS is the common technology for epigenomics analysis.

### 2.8 Microbiomics

Microbiomics is to study the ecological community of microorganisms that symbiotically or pathologically live on plants and animals, including bacteria, archaea, protozoa, fungi, and viruses.^60^ Due to the composition of the human microbiome being unique across individuals, the influence of the human genome on the gut microbiota is limited and generally influenced by the environment.^61^ Thus, the microbiome can be used to explain clinical variations in phenotypes of interest under specific conditions better than genetic factors in humans. For those characteristics of the microbiomics, many diseases have found a relationship with the gut microbiome, such as neurological disorders,^62^ renal failure,^63^ and cancer.^64^ The macro‐genome and macro‐transcriptome are the main technical tools for studying the microbiome, and 16S rRNA gene sequencing is the most commonly used method for microbial diversity analysis.^65^

### 2.9 Radiomics

In the medical field, radiomics refers to extracting high‐throughput image features from the region of interest of radiographic images (computed tomography (CT), magnetic resonance (MR), positron emission tomography [PET], etc.) and the precise quantification of lesion areas, and key information (such as biomarker) through machine learning (ML) methods,^66^ and ultimately aiding in the diagnosis, classification, or grading of diseases.^67^, ^68^ The radiomic features from PET imaging are better at predicting treatment response than conventional measures, such as tumor volume, diameter and metastases, and so on.^69^, ^70^ Deep learning (DL) of PET reconstruction and post‐processing of traditional reconstructed images can restore or reconstruct PET images of higher quality than traditional ordered‐subset expectation maximization through reducing image noise to obtain more details of radiation features.^71^ However, class imbalances and overfitting are common defects in radiomics, such as the low prevalence of certain diseases in the cohort results in an inability to distinguish between affected and unaffected lesion areas on PET images.^71^

## 3 EXPERIMENTAL DESIGNS AND CAUTIONS FOR MULTI‐OMICS

**FIGURE 2:**
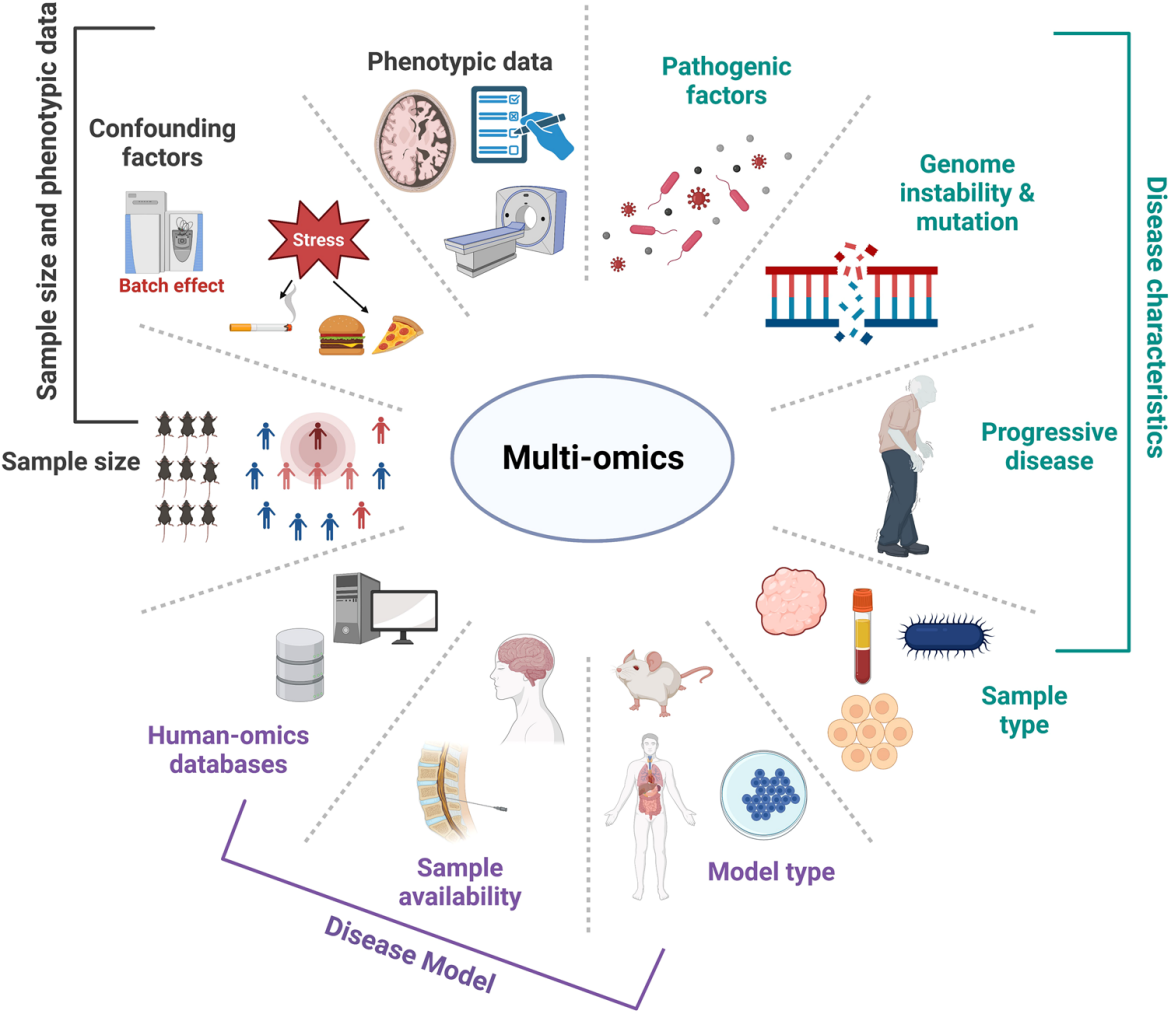
Notable recommendations for experimental design of multi‐omics. We list a lot of factors influencing the experimental results during experimental design, among them, three main factors include disease characteristics, choice of disease model, sample size, and phenotypic data. The disease characteristics contain pathogenic factors (bacteria or virus), genome instability and mutation, progressive disease (neurodegenerative disease), and sample type (tissue, cell, blood, or microbe). The disease model can be chosen according to suitable model type (human, mouse, or cell line), sample availability (unavailable samples, such as brain tissue and cerebrospinal fluid), and human‐omics databases (access to existing human disease omics data to compensate for uncommon disease models). The sample size and phenotypic data also need to be considered in multi‐omics experiment, such as the appropriate number of animal or human samples to achieve economically reliable omics results, while the appropriate number of detected samples usually according to confounding factors (batch effect, environmental stress like diet and smoking). The phenotypic data contain pathology, questionnaires, images, and so on.

Due to the high cost of multi‐omics technology in experiments, different types of omics have differences in sample collection time, collection conditions, and setting groups, and samples involving human sources are more precious, careful design of experiments is often required in multi‐omics studies. And detailed design will improve the repeatability and reliability of multi‐omics results, especially for large sample sizes, multiple comparison groups, and specific data analysis experiments.^6^ Moreover, the experimental design of reliable research is often inseparable from excellent statistical guidance, which can help to identify the research problem, study analysis, data interpretation, and conclusion.^72^ Here, we will discuss some notable captions for multi‐omics design (Figure 2).

### 3.1 Sample collection according to disease characteristics

Disease characteristics are important factors to consider in multi‐omics study design. Diseases caused by single‐gene mutations often have fewer pathogenic factors that play an important role in the development of the disease. For example, Duchenne muscular dystrophy is caused by a gene mutation of dystrophin, which helps keep muscle cells intact.^73^ The mutation of phenylalanine hydroxylase gene‐induced phenylalanine accumulated in the body that leads to phenylketonuria.^74^ For these types of diseases, collecting special tissue samples at specific time points and performing multi‐omics detection to deeply analyze the immediate molecular changes caused by pathogenic factors will help to enhance the understanding of the disease and find possible treatment strategies. Additionally, the pathogenic factors of some diseases also contain infectious agents, more than genetic mutations, such as Coronavirus disease 2019 (COVID‐19).

However, most diseases are caused by multiple factors rather than focusing on a single factor, and the pathogenesis is more complicated. Combinations of different factors may converge into phenotypically similar states. For these common and complex diseases, pathologic processes usually span a long time, and involve the interaction with genes, environment factors, and so on. Therefore, it is necessary to collect at multiple time points and different types of tissue samples for multi‐omics analysis, so as to reveal the mechanism of disease occurrence and development in depth. For example, in the case of Alzheimer's disease (AD), which has an uncertain etiology, multi‐omics studies often analyze AD patients or AD model mice at different ages and/or different types of samples (cerebrospinal fluid, plasma, or brain tissue).^75^, ^76^, ^77^

### 3.2 Establishment of the disease model (human or animal)

The main purpose of medical research is to solve human diseases, so human‐related omics research has more translational potential than animal model‐related omics research. Several human‐omics databases have been established, including epigenome and transcriptome databases of different tissues or cell types, such as the public database of IHEC Data Portal (https://epigenomesportal.ca/ihec/) and the Human Protein Atlas (https://www.proteinatlas.org/). Moreover, there have been some established large human disease repositories, collecting medical information and samples from different patients, such as the UK biobank.^78^

However, there also have some limitations in the study of human omics, and the replacement of animal disease models can solve these problems. For example, some human samples are not easy to collect, there are many confounding factors in human samples, and human‐derived cell lines cannot replicate the complex molecular network changes in vivo. While the animal model has the advantages of easy sample collection, uncontrolled sample size, clear phenotype, high reproducibility, easy environmental control, and so on. Animals play an important role in the research of diseases caused by environmental factors and can link omics data with corresponding environmental factors to reveal the pathogenesis of diseases. For instance, an animal model was used to study the effects of a high‐fat diet on nonalcoholic fatty liver disease.^79^ Furthermore, comparing omics data from human and animal models will help to validate the biological relevance of the models, such as mice as useful models for functional studies of AD gene regulatory regions.^80^ Nevertheless, animal model also has some application drawbacks, many transgenic models are restricted to one genetic background, some human disease manifestations cannot be reproduced in mouse models, and mouse models may not account for human biological processes in complex diseases.

### 3.3 Sample size and phenotypic data

The reliability of the information on omics results often depends on effect size, the sample size tested, and the magnitude of the effect of confounding factors. In terms of human studies, there have been many confounding factors, such as diet and lifestyle. Therefore, omics research on human diseases requires a large sample size to avoid misinformation and false‐positive results from individual abnormal samples. The sample size calculated from the initial efficacy is increasingly important for the reliability of the results of large‐scale studies.^81^ Moreover, omics experiments often overlook the need for data analysis before and during data collection, and most fields of omics have not yet developed a gold standard for data analysis. Thus, in order to ensure that the collected data meet the requirements of subsequent analysis, the design of omics research needs to carefully consider the main objectives of the experiment and the analysis method before collecting the data. For instance, in order to acquire important molecules that are closely related to phenotype, some critical phenotypic data need to acquire in omics, such as the data of histological features, tumor stage, smoking history, and mutation status need to be collected in the study of lung adenocarcinoma.^82^ The acquisition of phenotypic data of the disease is crucial for omics to reveal the molecular changes in the progression of the disease, and may directly find the key target molecules of the disease. For example, in the multi‐omics discovery of peripheral blood biomarkers of Alzheimer's disease, various cognitive impairment scale scores (MMSE and MoCA), brain imaging (PET and MRI), and other phenotype data play a decisive role in the discovery of diagnostic biomarkers.^83^, ^84^, ^85^, ^86^ Similarly, the purpose is to know the differentially expressed molecules between the disease and the control, so the experimental design should focus on the sample size^87^; if the experiment wants to find new molecules, the omics design should focus on a higher technical coverage depth, such as long‐read transcriptome.^88^ In addition, in order to avoid or minimize technically introduced errors, batch effects introduced in sample processing and data acquisition should also be considered in the design of omics experiments.^89^, ^90^

## 4 INTEGRATED ANALYSIS APPROACH FOR MULTI‐OMICS DATA

**FIGURE 3:**
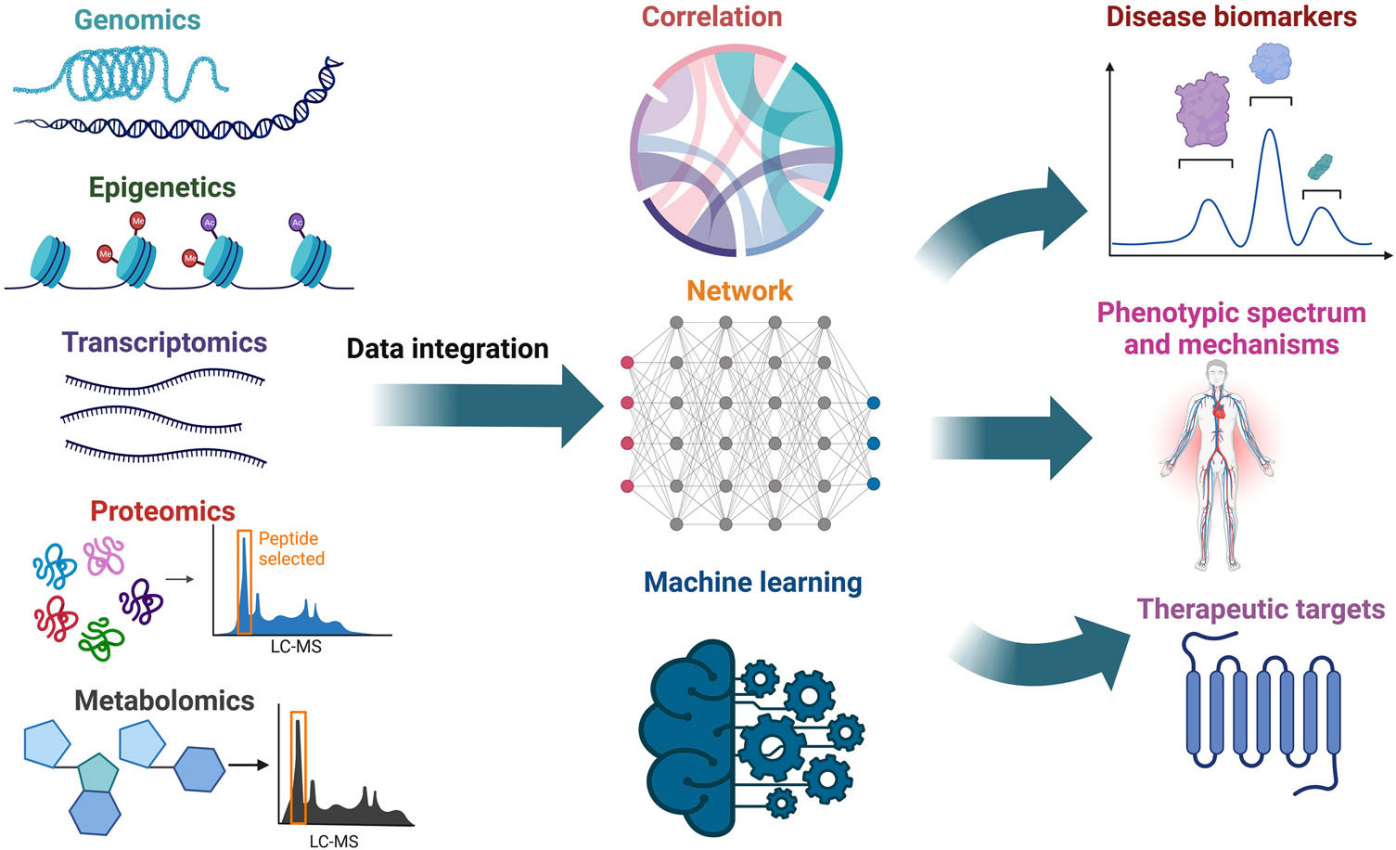
The methods for multi‐omics data integration. Here simply shows the multi‐omics data (such as genomics, epigenetics, transcriptomics, proteomics, and metabolomics) integration method based on the correlation of each omics, molecular network construction at different levels, and machine learning. The ultimate goal of data integration is to discover disease biomarkers, confirm phenotypic spectrum and mechanisms, and identify therapeutic targets.

Although the detection of multi‐omics can more deeply analyze the occurrence and development of diseases, and advance the development of systems biology compared with single omics, the integration of omics data has always been a cumbersome and nonstandard difficult problem. In most cases, multiple approaches to integrating omics data at multiple levels can be employed depending on the experimental design.^91^ Before the integration of multi‐omics data, we need to understand the characteristics of multi‐omics data. The multi‐omics data acquired from different technology are usually heterogeneous,^92^ such as transcriptomics and proteomics have different dynamic ranges and data distribution, due to the different normalization and scaling techniques among various omics analyses. In addition, some omics may produce null values due to being below the detection line of the instrument, such as metabolomics.^93^ Therefore, before integrating multi‐omics data, the imputation^94^ and outlier detection^95^ for each omics data should be considered separately. Next, we briefly discuss some current common methods for multi‐omics integration (Figure 3).

### 4.1 Mining correlation and network in multi‐omics data

After acquiring multi‐omics data, the fundamental analysis contains single omics annotation, biological information derived from single omics, and biological network between two or more omics data, such as gene and metabolite or protein (enzyme) and metabolite. However, the integration of multi‐omics data is the toughest problem in dealing with multi‐omics analysis. A relatively common and simple method for multi‐omics data integration is the correlation or co‐mapping between two different omics data. For example, there is a relatively direct correspondence between transcriptome and proteome, the integration of those two omics usually contains an analysis of molecules with consistent or different trends in differentially transcribed genes and differential proteins, and a correlation of expression level between transcribed genes and proteins.^96^, ^97^ Sometimes, there is also a need to compare the correlation of expression level between transcriptome and proteome, and many research results have shown that the correlation between the expression of mRNA and protein is not strong, indicating that the post‐transcriptional regulation may be involved, such as in Alzheimer's disease, which has been reported that 42% of Tau‐induced transcripts were discordant with the proteome, showing the opposite direction of change.^98^ Thus, the correlation between two omics will contribute to a better understanding mechanism in the process of disease occurrence and development.

In addition, the integrated multi‐omics also needs to take an effort to analyze the network between different omics data. The network serves as an established mathematical model for describing the complex interactions and regulatory mechanisms that occur in biological systems,^99^ such as protein−protein interaction.^100^ Network‐based approaches to the integrated analysis of multi‐omics data provide a framework to conceptualize the complex interactions of multi‐omics molecules as a collection of connected nodes (molecular features) in a network. Through these networks, it is possible to further identify associations (e.g., gene, protein, and metabolite relationships) and/or subnets (e.g., biological pathways) that can inform observed phenotypes.^101^ Compared with transcriptome and proteome, metabolome records the real biochemical reactions in biological processes, and measures the metabolic level of substrate and product in chemical reactions, while transcriptome and proteome measure and quantify enzymes that catalyze biological reactions. Therefore, in multi‐omics research with metabolomics, the molecular regulatory network between each omics data and metabolites is often analyzed. However, network construction is usually applied to a single omics, and appropriate methods still need to be developed for network interactions between multi‐omics.^102^ One simple and convenient approach to address multi‐omics network construction is to refer to public databases and frameworks, for example, omicsNet,^103^, ^104^ MetaboAnalyst,^40^ and ConsensusPathDB.^105^ Additionally, various algorithms (meta‐path‐based techniques,^106^ random walk,^107^ and module identification^108^) of heterogeneous networks can be used to build multi‐level complex networks, such as HENA,^109^ an Alzheimer's data set based on heterogeneous network, which integrated AD‐related co‐expression networks from public databases and experimental data sets to identify disease‐associated genes. Bodein et al. proposed to construct a hybrid multi‐omics network from longitudinal multi‐omics data using a random walk algorithm, highlighting key intra‐ and inter‐omics mechanisms and interactions.^110^ Overall, the integrated analysis of highly complex networks in multi‐omics is not straightforward, requiring complex algorithms, and the downstream functional interpretation and validation of multi‐omics findings is not straightforward either. Therefore, for most researchers, the construction of multi‐omics networks and the interpretation of the resulting data still require open‐source tools and shared databases, such as netOmics,^110^ MetaboAnalyst 5.0,^40^ MergeOmics 2.0,^111^ and so on (Table 1).

**TABLE 1:** The online tools and resources of network‐based multi‐omics knowledge bases.

Tool name	Biological entities	Implementation	Ref
netOmics	Genes, RNAs, proteins, metabolites	R package	^110^
HetioNet	Genes, SNPs, proteins, compounds, tissues, diseases	Online	^112^
MergeOmics 2.0	Genes, SNPs, RNAs, proteins, metabolites	Online	^111^
MetaboAnalyst 5.0	Genes, metabolites	Online	
MiBiOmics	Genes, RNAs, proteins, metabolites	Online	^113^
PathMe Viewer	Genes, proteins, metabolites	Online	^114^
OmicsNet 2.0	Genes, proteins, TFs, miRNAs, metabolites	Online	^115^
PathwayCommons	Proteins, metabolites, drugs	Online CyPath2^a^	^116^
Recon3D	Genes, metabolites	Online	^117^
BioCyc	Genes, proteins, metabolites	Online	^118^
PaintOmics3	Genes, proteins, metabolites	Online	^119^
MetExplore	Genes, enzymes, metabolites	Online	^120^

^a^ Applicable in Cytoscape software.

### 4.2 ML and DL for multi‐omics integration

**FIGURE 4:**
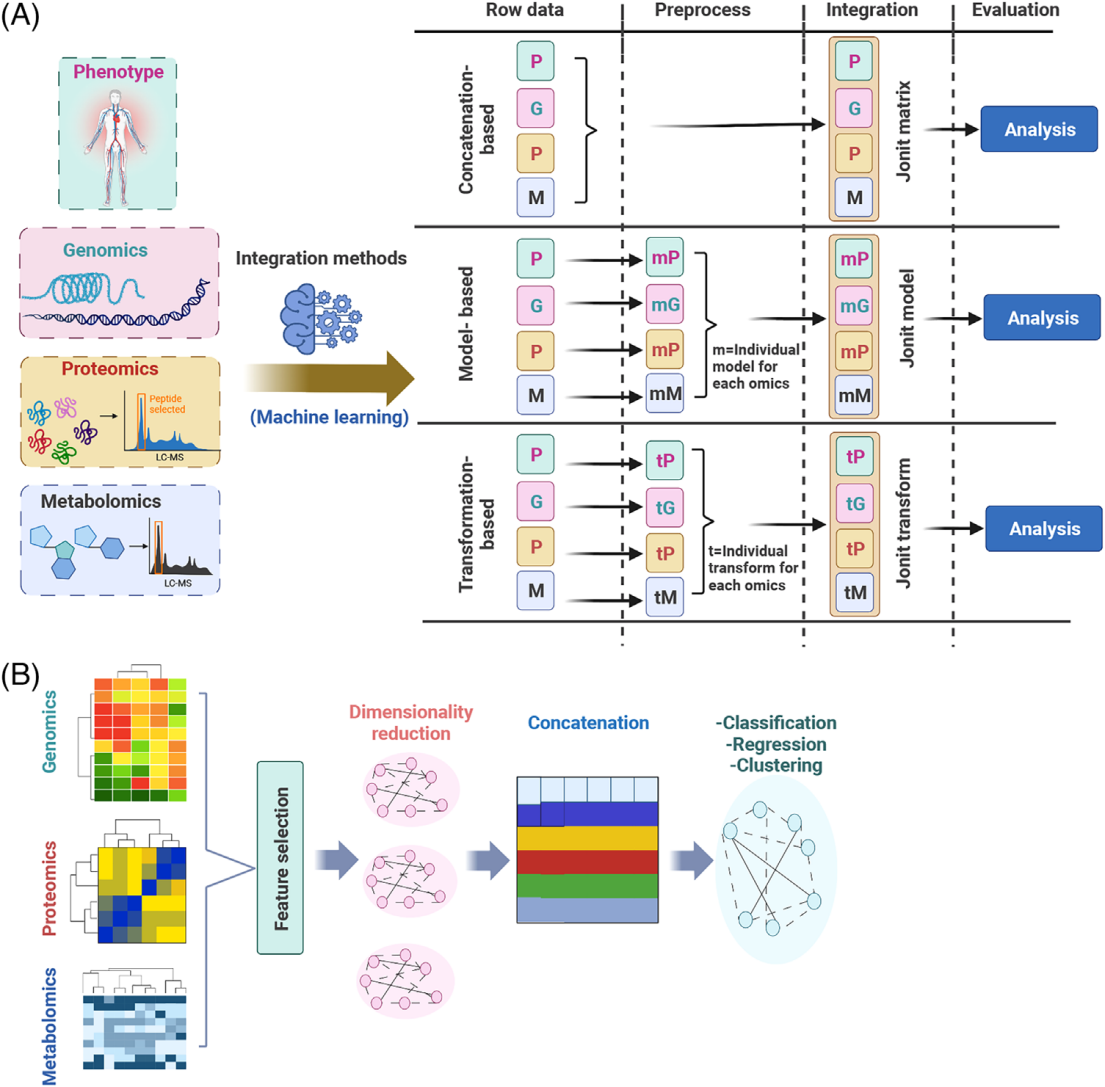
The brief process of integrating multi‐omics data with machine learning and deep learning. (A) The process of data integration by machine learning. The concatenation‐based integrated approach pipeline includes raw data from individual omics with corresponding phenotypic information, the data from the individual omics are then concatenated to form a single large matrix of multi‐omics data, and finally, supervised or unsupervised methods are used for joint matrix analysis. The model‐based integration method flow contains the establishment of the original data of various omics and the corresponding phenotypic information, develop individual models for each omics and then integrate them into a joint model, and finally, to analyze the joint model. And transformation‐based method starts with raw data of individual omics and corresponding phenotypic information, followed by developing individual transformations (in the form of graphs or kernel relations) for each omics, and then integrating it into joint transformations, and finally, analyzing it. The letters of PGPM are represented as phenotypic data (P), genomic data (G), proteomic data (P), and metabolomic data (M) in sequence. (B) The brief concept of data integration is achieved by deep learning. First, preprocess and clean the multi‐omics data, and then use conventional feature selection techniques or feature reduction methods for feature selection or dimensionality reduction to reduce the number of multi‐omics variables. Next, multiple omics variables are concatenated into one large data set for data integration. Finally, further feature selection or reduction techniques are applied to reduce the variables, and the integrated data are analyzed using classification, regression, and clustering.

With the development of high‐throughput omics technologies, multi‐omics data can also be integrated by ML‐ or DL‐based predictive algorithms to reveal the complex work of systems biology. ML is increasingly used in the development of precision medicine based on big data^121^, ^122^ and data mining,^123^ these techniques facilitate the discovery of new omics biomarkers that can identify the molecular causes of disease. However, unlike the correlation‐ and network‐based integration approach, the approach of using ML to integrate multi‐omics data has some unique challenges. First, ML may result in class imbalance and overfitting during disease classification and subsequent model training in multi‐omics data set.^124^ For example, the ML‐trained model using an imbalance data set, such as hypertension, which is only 5% of patients with endocrine hypertension, may be overfitted and lead to underperformance for unseen test data, despite high accuracy for training data. For this situation, it can be solved by collecting more data, or using weighted or normalized metrics (e.g., F1‐Score or Kappa^125^) to measure the ML performance, oversampling, or synthetic sample generation of under‐represented class. Second, the data sets of multi‐omics usually suffer the problem of “curse of dimensionality,” which refer to inconsistency in data dimensions and features,^126^ leading to redundant features in high‐dimensional space followed by misleading algorithm training. While, feature extraction and selection can be used for dimensionality reduction, such as principal component analysis (PCA), linear discriminant analysis, biofilter, and so on.^127^ Third, the appropriate algorithms are essential for multi‐omics analysis, and previous reviews reported that different ML algorithms have diverse strengths and weaknesses in integrating multi‐omics data sets derived from cancer‐related research.^128^ Reel et al. have proposed a flowchart that can help the interdisciplinary user to choose the appropriate algorithms from available methods.^129^ Finally, ML requires high computational power and large storage for data processing and analysis, while the corresponding cost should be considered before planning an ML‐based multi‐omics workflow. Moreover, reasonable transparency and explainability are also important for multi‐omics integration by ML, and thus can be helpful for building trust in clinical decision‐making.^130^ Therefore, when using ML to integrate multi‐omics data, researchers should consider all the above direct influences. Next, we will briefly introduce three ML‐based multi‐omics integration methods from the published reports,^129^, ^131^ which are shown in Figure 4A.

#### 4.2.1 Concatenation‐based integration methods

Concatenation‐based integrated approach pipeline includes raw data from individual omics with corresponding phenotypic information, and then, the data from the individual omics are concatenated to form a single large matrix of multi‐omics data, and finally, supervised or unsupervised methods are used for joint matrix analysis. Specifically, multiple data matrices for each sample in multi‐omics were combined into a large input matrix, and then different classical ML methods are used for data analysis. For example, data from gene expression, copy number variation, and mutation were combined into a joint matrix, and the joint was then used with classical random forest and SVM (support vector machine) to predict anticancer drug response.^132^ A joint matrix of multi‐omics features (which included SNPs and mRNA gene expression) was used with the Bayesian integrative model to assess drug cytotoxicity.^133^ According to the characteristics of ML, the concatenation‐based integration methods can be classed as unsupervised and supervised type. A variety of concatenation‐based unsupervised methods are available for clustering and association analysis, such as MoCluster, which can find the joint clusters among inputted multi‐omics data.^134^ The Multi‐Omics Factor Analysis (MOFA) can disentangle the heterogeneity shared across different omics to discover the principal source of variability.^135^ The concatenation‐based supervised learning methods that usually can be used for phenotypic prediction, such as boosted trees and SVR, have been investigated for identifying longitudinal predictors from a large multi‐omics data set of type 2 diabetes.^136^ Advantages of concatenation‐based integration methods include that continuous or categorical data can be easily analyzed using ML, and the most discriminative features can be selected for a given phenotype. However, there has a challenge in concatenation‐based integration for combining multiple matrices that include data from different scales. For instance, the value of SNP data contains 0, 1, or 2, while the value of copy number data may consist of −2, −1, 0, 1, or 2, and the value of CpG loci between 0 and 1. Thus, concatenating this form of data integration can inflate high‐dimensionality during data analysis, and concatenation‐based integration is only possible applied after performing data reduction.

#### 4.2.2 Model‐based integration methods

Model‐based integration is a method of generating multiple models by using different types of data as training sets, and then generating a final model from the multiple models created in the training phase. Specifically, this integration method contains: (1) establishment of the original data of various omics and the corresponding phenotypic information; (2) development of individual models for each omics and then integrate them into a joint model; and finally, (3) analysis of the joint model. This approach can combine predictive models from different omics and facilitate the understanding of a certain phenotype among different types of data. For example, to identify genetic, genomic, and proteomic associations with ovarian cancer, model‐based integration will allow independent analysis of each of the DNA sequence data, microarray data, and proteomic data, and then integrate top‐level models from each data set to find integrated models, such as model‐based integration performed with ATHENA (Analysis Tool for Heritable and Environmental Network Associations) to create an integrative model from each data type of ovarian cancer.^137^ Similarly, the model‐based integration methods also divide into unsupervised and supervised types. Various model‐based unsupervised learning methods have been achieved, such as patient‐specific data fusion, which is used for clustering of predicted cancer subtypes by combining gene expression and copy number variation data,^138^ perturbation clustering for data integration and disease subtyping^139^ and Bayesian consensus clustering^140^ are more flexible and allow late‐stage integration of clusters. Supervised learning model‐based methods include various frameworks for developing models, such as Multi‐omics Supervised Autoencoder, which is used for pan‐cancer analysis,^141^ DFNForest (hierarchical integration deep flexible neural forest) framework integrated three omics data sets to predict cancer subtype classification,^142^ and so on. An advantage of model‐based integration approaches is that they can be used to combine models based on different omics types, where each model was developed from a different patient group with the same disease information. However, model‐based integration also needs specific hypothesis and analysis for each data type, and appropriate mechanisms for the resultant model combination. When integrating models constructed from different types of data, some interactions between different data types may be missed because the only variables are those detected during the data type‐specific modeling process. For instance, the methylation pattern and another protein expression pattern that are not independently associated with the results, but only through their interactions, their effects will be missed in the model‐based integration. Thus, model‐based integration is particularly suitable if each genomic data type is very heterogeneous.

#### 4.2.3 Transformation‐based integration methods

Transformation‐based integration involves converting each omics data set into an intermediate form (such as a graph or kernel matrix), and then combining them together before elaborating any models. Briefly, this method starts with raw data of individual omics and corresponding phenotypic information, followed by developing individual transformations (in the form of graphs or kernel relations) for each omics, and then integrating it into joint transformations, and finally analyzing it. The graph‐based method provides a formal method for transforming and portraying relationships between samples of different omics. The kernel‐based method transforms the data from the original space to a higher dimensional feature space. These methods then explore linear decision functions in the feature space that are nonlinear in the original space. Various transformation‐based unsupervised methods have been introduced, such as PAMOGK, which can integrate multi‐omics data with pathways,^143^ somatic mutations, transcriptomics, and proteomics data were used to identify subtypes of kidney cancer. While transformation‐based supervised learning methods are mostly kernel‐based and graph‐based algorithms, such as Multi‐Omics gRaph cOnvolutional NETworks, which uses graph convolutional networks to take advantage of both the omics features and the correlations among samples described by the similarity networks for better classification performance.^144^ A major advantage of transformation‐based integration methods is that they can be used to combine broad omics studies if unique information (patient ID) is available. The disadvantage of transformation‐based integration is that each data type is transformed independently, which makes it more difficult to detect interactions between different types of data, such as an SNP and gene expression interaction. Therefore, conversion‐based integration is suitable for each data type with an associated intermediate representation, such as a kernel or a graph.

In short, the above‐described approaches for multi‐omics data sets integration are based on various supervised and unsupervised ML methods. The advantages and disadvantages of using those integrative methods, and the detailed various multi‐omics integration methods based on ML can be found in the previous review.^129^

Moreover, DL has emerged as one of the most promising approaches in the integrated analysis of multi‐omics data due to its predictive performance and ability to capture nonlinear and hierarchical features. DL can integrate different omics data from clinical or health records with high specificity, sensitivity, and efficiency,^145^ and has the ability to automatically capture nonlinear and hierarchical representative features with multi‐layer neural network architecture, which has an excellent performance in prediction. For example, the multi‐omics integration approach with DL can robustly predict liver cancer survival,^146^ and the DL approach can predict AD on the basis of combined neuroimaging and genomics data.^147^ Overall, most DL‐based data integration studies are categorized into clustering for feature selection/reduction, clinical outcome prediction, survival analysis, and subtype discovery. For these studies, the DL models for multi‐omics data integration analysis follow a general principle^148^ (Figure 4B). First, preprocess and clean the multi‐omics data, and then use conventional feature selection techniques or feature reduction methods (such as PCA and autoencoder) for feature selection or dimensionality reduction to reduce the number of multi‐omics variables. Next, multiple omics variables are concatenated into one large data set for data integration. Finally, further feature selection or reduction techniques are applied to reduce the variables, and the integrated data are analyzed using classification, regression, and clustering. For example, in the survival analysis study of liver cancer reported by Chaudhary et al.,^146^ first, transforming features of omics data (mRNA, DNA methylation, and miRNA) using a DL framework (autoencoder) to generate new features. Second, the univariate Cox‐PH model was used for identifying survival‐associated features, which then were clustered by a K‐means clustering algorithm to identify cancer subtypes. Third, according to the cluster labels obtained from K‐means to build supervised classification model(s) using the SVM algorithm, and data samples were divided into training sets and test sets followed by fivefold cross‐validation to find the best hyperparameters of the SVM model(s). Finally, concordance index (C‐index), log‐rank *p*‐value of Cox‐PH regression, and brier score were used for models’ evaluation, and then, the acquired model to predict the survival risk labels of new data sets. In addition, many DL tools for integrated analysis of multi‐omics data have been developed, such as DeepOmix, DeepProg, and DeepDRK. DeepOmix can be used not only for survival prediction analysis, but also for predicting various clinical indicators, such as drug response.^149^ The DeepOmix integrated different omics data as input gene layer, and connected gene layer nodes to functional layer according to the prior information of pathway or functional module defined by input. The basic idea behind the model is that the gene function module layer is introduced into the algorithm model, and the biological prior information of the gene module is fused to integrate the multi‐omics characteristic information from the sample, and then applied to the prediction of survival state. The training model can predict the survival period of patients, and obtain the low‐dimensional representation of sample data in the functional module layer. Through statistical analysis, the functional modules of genes affecting prognosis can be found. DeepProg is used to integrate multi‐omics phenotypes, such as survival to predict cancer prognosis.^150^ The method can be briefly described as follows: first, the custom rank normalizations and auto‐encoders were used for dimension reduction and feature transformation, second, the univariate Cox‐PH fitting was performed on the transformed features to further select the feature subset related to survival, finally, using an unsupervised clustering approach to identify the optimal number of classes (labels) for survival subgroups followed by building a support vector machine (SVM)‐based ML model according to these classes, and then using the acquired model to predict survival groups for new patients. DeepDRK predicts cancer cell drug response by integrating pharmacogenomics data sets, providing an alternative approach to prioritizing drug repurposing in precision cancer therapy.^151^ DeepDRK used a kernel‐based approach to generate integrated representations of CCL and anticancer drug interaction partners, which were then employed to train deep neural networks for drug response prediction. Briefly, various types of multi‐omics data were collected, and CCL similarity matrices based on multiple kernels were constructed, respectively, then two similarity matrices for anticancer drugs were calculated using chemical characteristics of compounds and drug−target interactions. The CCL−drug pairs were represented by concatenating multiple similarity vectors, and then training the classification model to predict drug efficacy. In addition, there also has another new method of DL for multi‐omics integration. For instance, Multi‐omics Attention Deep Learning Network focuses on the correlations between patients and omics.^152^ Specifically, the authors used three fully connected layers to reduce dimensionality and extract the significant features from omics data of mRNA, DNA methylation, and miRNA, and used the self‐attention (SA) mechanism to construct the correlations between patients, respectively, for omics‐specific feature learning. The initial category labels were generated using the feature vectors learned from the SA. Then, the Multi‐Omics Correlation Discovery Network was used to learn the cross‐omics correlations in the label space for the initial label predicted of each omics data. Finally, the SoftMax classifier was used for label prediction.

However, like ML, DL also encounters challenges, such as high‐dimensional and low sample‐size data, missing data and data heterogeneity, model interpretability, and integrating clinical and environmental exposure data. In general, the method of multi‐omics data integration will become easier and more feasible for nonprofessionals to obtain more biological information through multi‐omics technology with the development of various computing frameworks and tools.

## 5 APPLICATIONS OF MULTI‐OMICS IN HUMAN DISEASES

**FIGURE 5:**
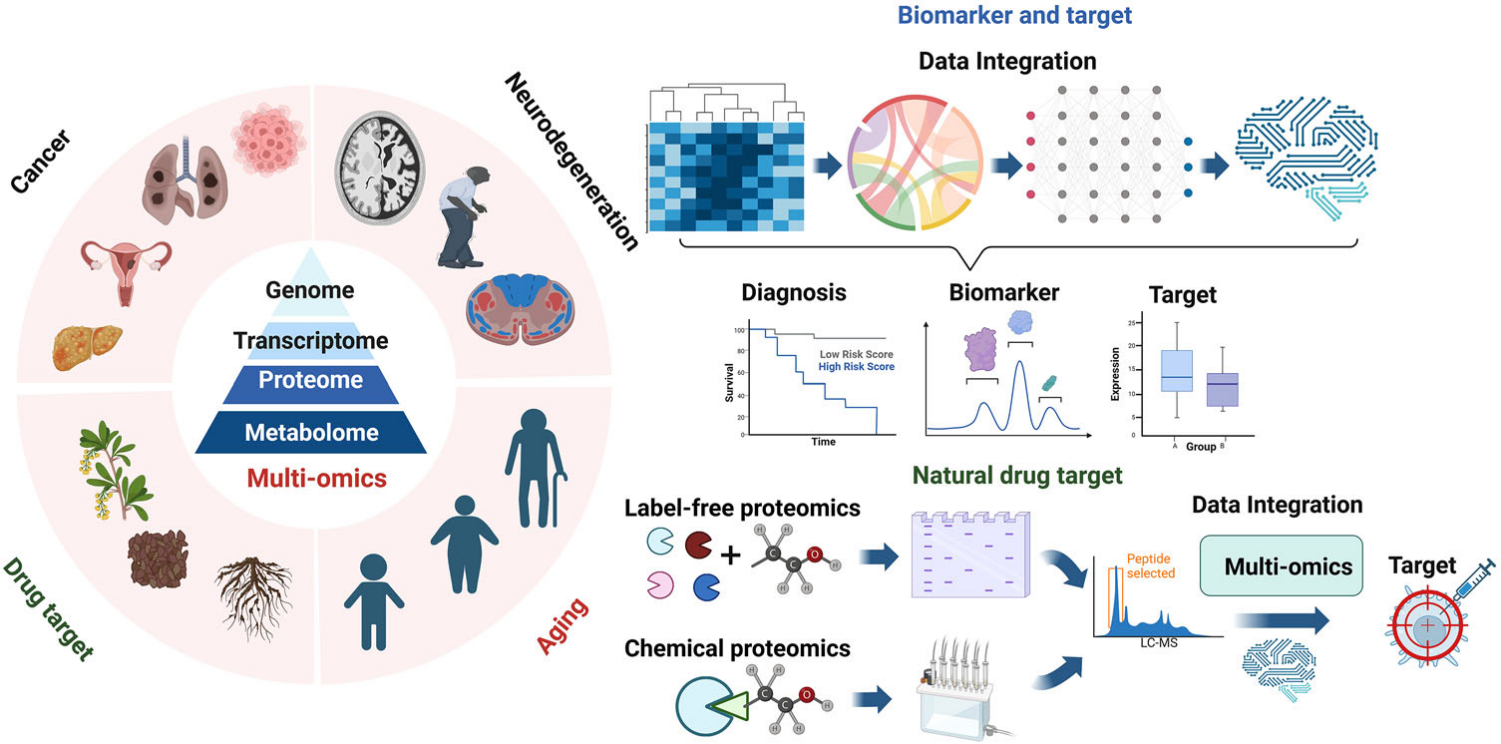
The application of multi‐omics in disease, aging, and natural drug target identification. There lists some disease‐associated applications of multi‐omics in cancer (liver cancer, lung cancer, ovarian cancer, and malignant lymphocytic tumor) and neurodegeneration (AD, PD, and ALS). Other applications, such as aging and natural drug (natural compound obtained from the flowers, seeds, or rootstock of plants) target screening, are also shown in the figure. The brief process and methods for the application of multi‐omics in their research area are listed right. For biomarker and target research, multi‐omics data are analyzed by differential expression analysis, correlation analysis, and network construction, and then, machine learning was used to obtain diagnostic biomarkers or therapeutic targets. The procedure for screening natural drug target is usually based on the integration of label‐free proteomics and chemical proteomics, and then is used for machine learning to acquire possible functional target.

In addition to the omics types, multi‐omics experimental design, and multi‐omics data integration analysis described above, we will introduce various applications of multi‐omics in human diseases, such as cancer (malignant tumor, lung cancer, liver cancer, and ovarian cancer) and neurodegeneration (Alzheimer's disease, Parkinson's disease, and amyotrophic lateral sclerosis [ALS]). Moreover, multi‐omics have also application in drug target discovery and aging research (Figure 5). The next part will introduce some previous studies of multi‐omics in human diseases.

### 5.1 Disease biomarkers and targets

Based on the outstanding advantages of multi‐omics in the discovery of disease etiological mechanisms, disease research increasingly requires the participation of multi‐omics and computational algorithms. Biomarkers can not only explore the pathogenesis at the molecular level, but also have unique advantages in accurately and sensitively evaluating early, low‐level damage, providing early warning, prognostic efficacy analysis, and accurate staging and typing of diseases. For clinical diseases, the discovery of disease biomarkers plays a guiding role in the diagnosis and prognosis of diseases.^152^ While the screening of biomarkers usually requires the use of high‐throughput omics methods to measure large‐scale clinical samples, followed by screening to statistically significant differential molecules (genes, proteins, or metabolites), and finally a series of complex bioinformatics analyses to screen out the target biomarkers. Although many cancer‐related biomarkers have been identified by single omics, multi‐omics is more advantageous for cancer research. Proteomics data reveal overlapping but not identical correlations with transcriptomic and genetic data in breast cancer,^153^ high‐grade serous ovarian cancer,^154^ or rectal cancer,^155^ and this phenomenon may account for the unique genetic and transcriptional process of proteomic alterations in the cancer process. For example, phosphoproteomic analysis of breast cancer identified G protein‐coupled receptor clusters which cannot be readily identifiable at the mRNA level.^153^ Integration of various omics information is expected to be valuable in guiding targeted cancer therapy.^156^ Moreover, there have been many successful examples in finding cancer biomarkers by multi‐omics, for example, diffuse large B‐cell lymphoma,^157^ ovarian cancer,^158^ and pancreatic cancer.^159^ In addition to the application in the discovery of tumor or cancer‐related disease biomarkers, multi‐omics is also applied to find biomarkers for other diseases, such as stroke,^160^ obesity,^161^ cardiovascular diseases,^162^, ^163^ severe COVID‐19,^164^, ^165^, ^166^ Alzheimer's disease,^167^ diabetes,^168^ obstructive sleep apnea,^169^ and so on.

**FIGURE 6:**
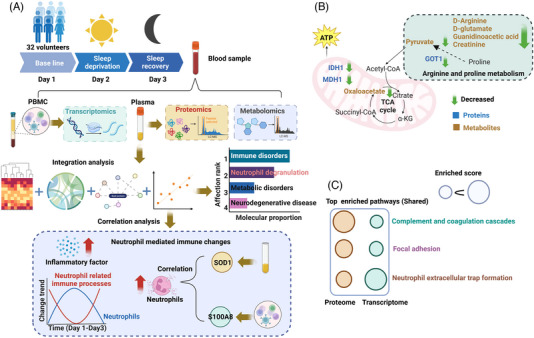
The integrated multi‐omics analysis on sleep deprivation. (A) The brief procedure and data for multi‐omics study in sleep deprivation. The blood sample obtained from 32 volunteers suffered sleep deprivation and recovery for plasma proteomics and metabolomics, PBMC transcriptomics analysis. After multi‐omics integration, the prominent biological pathway induced by sleep deprivation was immune disorders followed by metabolism disorders and neurodegenerative disease, and neutrophil degranulation was main account for the immune change. The detailed analysis showed that immune cell counts and inflammatory factor levels were elevated, and neutrophils and their mediated immune processes were highly coordinated with sleep states. The correlation analysis revealed that SOD1 and S100A8 were the most likely biomarkers of sleep deprivation‐induced immune changes. (B) The integration of proteomics and metabolomics, and the differentially expressed proteins and metabolites linked the pathway of arginine and proline metabolism, the TCA cycle. (C) The integration of proteomics and transcriptomics, and the shared top pathways enriched by differentially expressed genes and proteins are listed.

Integrated omics can also be used to investigate the influence of environmental factors on early disease formation in humans, such as sleep deficiency. Our study integrated analysis of transcriptomics, proteomics, and metabolomics in the blood of young health people suffering from transient sleep deprivation^170^ (Figure 6). In this study, 32 volunteers suffered 1 day of sleep deprivation, and donated fasting blood samples prior to (Day 1) and following (Days 2 and 3) short‐term sleep deprivation. Then, the plasma was used for proteomics and metabolomics analysis, and the peripheral blood mononuclear cell (PBMC) was used for transcriptomics analysis. After integration analysis, the prominent biological pathway was immune disorders. Further detail analysis found that neutrophil‐mediated immune processes (such as neutrophil degranulation) mainly account for sleep deprivation‐induced immune disorders. And the correlation analysis showed that SOD1 and S100A8 may be served as biomarkers for immune disorder caused by sleep deprivation (Figure 6A). Noteworthily, the integrated analysis of metabolomics and proteomics showed that differentially expressed metabolites and proteins were involved in pathways of arginine and proline metabolism, tricarboxylic acid cycle (TCA) cycle has a similar trend of changes, and pyruvate and GOT1 linked these two pathways (Figure 6B). In addition, the integrated pathway analysis of proteome and transcriptome showed that differentially expressed genes and proteins shared the highest enriched score of pathways (such as neutrophil extracellular trap formation, complement and coagulation cascades, and focal adhesion) (Figure 6C).

Compared with cancer or chronic diseases (diabetes), neurodegeneration acts as a major disease and lacks effective treatments, multi‐omics has important applications in the discovery of diagnostic biomarkers, pathogenic mechanisms, and therapeutic targets for neurodegenerative diseases. In view of the increasing aging of the population and the lack of effective treatments for neurodegenerative diseases, the next section will focus on the application of multi‐omics in neurodegenerative diseases, which were represented by AD, PD, and ALS, with special emphasis on the discovery of diagnostic biomarkers and therapeutic targets.

### 5.2 Applications of multi‐omics in neurodegenerative diseases

As the most common neurodegenerative disease, the prevalence of AD has been increasing year by year. A global public health study estimated that the number of AD patients will increase from the current 57 million in 2019 to about 150 million in 2050,^171^ which leads to significant increases in medical and social costs.^172^ However, there is still no effective treatment for AD, and the pathogenesis of AD is still not fully understood, although amyloid (Aβ) plaques and tau neurofibrillary tangles have revealed major pathological changes in AD.^173^ Therefore, a comprehensive understanding of the molecular mechanisms underlying AD pathophysiology by multi‐omics will help to provide support for the prevention, treatment, and prognosis of AD.^174^ Systematic integration of multi‐omics disciplines, including genome, transcriptome, proteome, and metabolome, will comprehensively and systematically reveal the pathophysiology of AD.^175^ Genomics, the study of describing and quantifying all genes and mutations in an organism, can identify new loci that increase AD risk and is critical to understanding the underlying causes of AD,^176^ such as high‐risk mutated genes of APP, PS1, Tau, APOE4, and so on. Transcriptomics, as a powerful approach to studying gene regulatory mechanisms, can map co‐expressed genomes of transcriptional programs associated with AD phenotypes.^177^ Proteomics explores proteins that play a role in AD by studying protein expression, protein−protein interactions, and post‐translational modifications.^178^ The changes of hippocampal proteins involved in insulin signaling and mitochondrial electron transport chain may be the crucial biological processes in AD progression.^179^ In our laboratory, by integrated hippocampal proteomics and phosphoproteomics, abnormal phosphoration of GSK3β and Ppp3ca was found to induce mitochondrial dysfunction in low‐dose copper‐treated AD mice.^180^ Yu et al. conducted platelet proteomics in patients with cognitive impairment in type 2 diabetes mellitus and reported that optineurin can act as a biomarker for predicting a cognitive decline,^83^ and also found that PHB, UQCRH, GP1BA, and FINC may be the most promising platelet biomarkers for predicting cognitive decline in mild cognitive impairment and AD patients.^84^ Metabolomics studies all the metabolites in the biological process of cells, which can reveal the persistent pathological process of AD,^181^ for example, sphingolipids may act as biomarkers for early AD by metabolomics.^182^

However, although signal omics can more or less provide information on the biological processes of AD progression, the regulatory and causal relationships among different levels of genes, mRNAs, regulatory factors, proteins, and metabolites are still unclear. For instance, although studies of the metabolome can be used to measure changes in biochemical pathways associated with AD pathogenesis, the relationship between systemic abnormalities of metabolism and AD pathogenesis is poorly understood, whereas integrating genetic, transcriptomic, metabolomic, and proteomic data in AD can enable the identification of AD‐specific metabolomic changes and their potential upstream genetic and transcriptional regulators.^183^ Therefore, integrated multi‐omics research can explore the entire biological continuum, and may find key molecules in the progression of AD. Comprehensive and in‐depth multi‐omics research, which integrates multi‐level biological information and explains the interaction between components, is consistent with the concept of systems biology.^184^ Multi‐omics analysis identified IVD, CYFIP1, and ADD2 as autoantibody biomarkers for distinguishing AD from controls,^185^ biomarkers (FBP1, FBP2, RHOH, JPH2, ERAp2, and SCLT1) distinct from AD patients with APOE2 and APOE4,^186^ ABCA1, CPT1A, adiponectin, and NGAL to be associated with AD pathology,^183^ and 14‐3‐3 zeta/delta and clusterin associated with AD and cognitive decline.^187^ Moreover, AD‐related multi‐omics data integration tools have been developed for the discovery of AD biomarkers and drug therapeutic targets, for example, Genome‐wide Positioning Systems Platform for Alzheimer's Drug Discovery (AlzGPS), which is used for the excavation of AD‐related targets and clinical‐related candidate drugs.^188^ Another platform based on gene regulatory networks can integrate multi‐omics data to identify key disease pathways and driver genes.^189^ Wang et al. obtained AD drug targets and biomarkers through a comprehensive analysis of multi‐omics data and animal genome‐scale metabolic model, which could eventually be validated and transformed into therapeutic or diagnostic methods.^190^ Collectively, identifying the associations of AD‐related brain functional and structural changes by integrating multi‐omics studies will contribute to a more comprehensive molecular understanding of the disease pathophysiology.^191^, ^192^

For Parkinson's disease (PD), the second largest neurodegenerative disease in the world, its main clinical manifestations are resting tremors and movement disorders, accompanied by some mental problems, such as cognitive impairment and depression.^193^, ^194^ However, the early diagnosis of PD has been a difficult problem in clinical medicine, the research on the etiology and biomarkers has a very important medical value for early diagnosis. In terms of a single omics study, through GWAS analysis, genes closely related to PD have been screened, such as SCNA, MAPT, and LRRK2.^195^, ^196^ Zhang et al. showed that SSR1 (the signal sequence receptor subunit1) can be used as a biomarker for the early diagnosis of PD by analyzing the transcriptomics of peripheral blood of PD patients.^197^ Proteomic analysis of cerebrospinal fluid of PD patients showed that OMD, CD44, VGF, PRL, and MAN2B1 were significantly correlated with PD clinical scores.^198^ Metabolomic study of sebum shows that sebum can be used as a biomarker for PD diagnosis.^199^ In the integration of multi‐omics, an integrated study of the PD gut microbiome and metabolome revealed that low short‐chain fatty acids were significantly associated with poorer cognition in PD, and lower butyrate levels were associated with worse postural instability gait disorder scores.^200^

Finally, for ALS which is characterized by loss of upper and lower motor neurons, multi‐omics technology has also been applied to study biomarkers for its diagnosis and prognosis.^201^, ^202^ For instance, a multi‐omics integration approach not only identifies neurotrophic factors and histamine as potential therapeutic targets, but also provides additional guidance for the personalized medical applications for ALS.^203^ GWAS have reported highly pathogenic mutations in ALS genes, such as C9ORF72, FUS, OPTN, SOD1, TARDBP, TBK1, and TDP‐43.^204^ ST of SOD1‐G93A transgenic mice reveals sphingomyelin signaling as a therapeutic target for ALS.^205^ UCHL1, MAP2, and GPNMB are reported to be promising biomarker candidates for ALS using proteomic studies of cerebrospinal fluid and spinal cord.^206^ Patin et al. reported that arginine and proline metabolism may be used as targets for ALS therapy through muscle and brain metabolomics studies in SOD1‐G93A transgenic mice.^207^ Moreover, omics has also reported many possible therapeutic targets or biological processes for neurodegenerative diseases. We reported that Mucolipin‐1 may be the target which can ameliorate mitophagy defect in 5×FAD mouse,^208^ and confirmed that TrkB receptor agonist R13 promoted mitochondrial biogenesis and function in 5×FAD and SOD1‐G93A mouse by upregulated PGC‐1α, NRF1, and TFAM by mitochondriomics.^209^, ^210^

Overall, multi‐omics has many applications in the study of neurodegenerative biomarkers and therapeutic targets. It is believed that with the development of high‐throughput histology technologies and integration algorithms, multi‐omics technologies will reveal more in‐depth molecular network changes in neurodegenerative diseases.

### 5.3 Applications in aging research

In addition to the application in the basic and clinical research of disease, multi‐omics is also being explored in aging science. Aging is a time‐dependent physiological process characterized by DNA mutations, epigenetic alterations, abnormal protein aggregation and autophagy disturbances, immune impairment, mitochondrial dysfunction, telomere shortening, abnormal intracellular signaling, nutrient‐sensing obstacle, and so on.^211^ These changes impair the normal function of cells and contribute to the development of age‐related diseases. And since aging is not only the greatest risk factor for many chronic diseases, but also a major cause of functional decline, there is a need to develop methods to measure the rate of aging in a given individual. The study of aging can be addressed by systematically describing the relationship between organ biomarkers, phenotypes (molecular biomarkers), and clinical presentation.^212^ A major and future trend in the field of aging is the development of omics‐based biomarkers, and these biomarkers have greater potential for assessing multifactorial processes. Over the past few years, genes, gene products, epigenetic modifications, and/or metabolites during aging have been profiled by high‐throughput sequencing, mass spectrometry, and other techniques. Aging is a very complex process that is influenced by a myriad of factors in addition to genetic predisposition, and the extent to which genes influence variation in the human aging process remains controversial.^213^, ^214^

In particular, genomics analysis of centenarians provides insights into genetic susceptibility to exceptional longevity.^215^, ^216^ Human lifespan GWAS of more than 500,000 participants proved that previous research found that APOE, FOXO3,^217^ and 5q33.3^218^ are longevity genes, and proposed five new genes related to longevity heritability.^219^ As epigenetic modifications are highly variable over the life cycle and potential biomarkers in response to aging, epigenomics shows a slow decline in total DNA methylation levels with age, and cytosine methylation containing CpG sites is hypermethylated or hypomethylation at different genomic locations with age.^220^, ^221^ The transcriptome is markedly altered during aging, and age‐associated transcriptome changes are highly tissue‐specific.^222^ Mamoshina et al. identified tissue‐specific biomarkers of aging through transcriptomic analysis of muscle tissue using ML algorithms.^223^ Plasma proteomic studies of healthy humans of various ages have found biomarkers highly correlated with age, such as growth differentiation factor 15.^224^ Lehallier et al. analyzed the plasma proteomics of 4263 healthy people aged 18−95, and revealed specific changes in the proteome in the fourth, seventh, and eighty decades.^225^ Compared with other omics, metabolomics has strong advantages in the sensitivity and predictability of the physiological state of the body in aging research.^226^ Previous studies have reported significant changes in plasma concentrations of a large number of metabolites correlated with aging.^227^ In a plasma metabolome study of a large cohort of men and women followed by up to 20 years follow‐up, higher concentrations of isocitrate and the bile acid taurocholate were associated with lower odds of longevity.^228^ However, single‐omics studies do not systematically account for the deep relationships between genes, proteins, metabolites, and other molecules in the aging process. For instance, epigenomics alone cannot reveal how epigenetic changes specifically regulate transcriptional changes and gene expression, whereas integrating data from transposase‐accessible chromatin (ATAC‐seq), RNA‐seq, and ChIP‐seq can identify major regulatory elements and key genes of aging.^229^ Through the integrated application of multi‐omics, molecules closely related to aging have been discovered, for example, transcription factors E2F4, TEAD1, and AP‐1 have been found to be key factors regulating aging by integrating transcriptome and epigenome.^230^ Song et al. revealed that NAT1, PBX1, and RRM2 may be potential biomarkers of aging and aging‐related diseases by integrating ATAC‐seq, RNA‐seq, and ChIP‐seq in a multi‐omics analysis.^229^ In order to facilitate the research of big aging‐related omics data, a multi‐omics database of aging biology has also been established, such as Aging Atlas, which provides user‐friendly functionalities to explore age‐related expression changes in genes, RNA, proteins, and so on.^231^ At the same time, it has also developed a multi‐omics data analysis platform and comprehensive database that provide aging‐related data in multiple species, such as AgingBank, which provided experimentally supported multi‐omics data relevant to aging in multiple species.^232^

### 5.4 Multi‐omics in the discovery of natural drug targets

Natural products are capable of selectively and specifically interacting with myriad molecular targets due to their complex molecular frameworks and diverse biological activities.^233^ Biologically active natural products often readily penetrate cell membranes and disrupt the physiological levels of the genome, transcriptome, proteome, and metabolome. These phenomena indicate that natural products have interactive properties with specific molecular targets in organisms. The discovery of new drug targets or new pharmacological effects of drugs is of great significance for expanding the scope of drug indications.^234^ Therefore, the discovery of precise drug targets is crucial to the development of new drugs. Significant progress has been made in drug target discovery through chemogenomics, chemoproteomics, label‐free proteomics, and bioinformatics approaches.^235^, ^236^ However, any single omics approach is insufficient to clearly define precise molecular targets in complex physiological networks, while an integrated multi‐omics approach can simultaneously elucidate, define, and validate multiple potential targets and mechanisms of candidate natural products.^237^, ^238^ For example, a single chemical proteomics strategy can obtain 14 possible targets of perfluorooctanoic acid (PFOA)‐induced toxicity and carcinogenic potential in humans; however, only the simultaneous combination of targeted metabolomics enabled the identification of acetyl‐CoA carboxylase 1 (ACACA, ACCa) and acetyl‐CoA carboxylase 2 (ACACB, ACCb) as bona fide binding targets of PFOA.^239^ Using genomic and proteomic (DARTS, drug affinity responsive target stability, a method to identify targets of small molecules^240^) integration, the natural product ecumicin was identified to inhibit *Mycobacterium tuberculosis* by targeting ClpC1.^241^ The natural product baicalin improved diet‐induced obesity and hepatic steatosis through carnitine palmitoyltransferase 1 (CPT1), which was also discovered through chemical proteomics and bioinformatics (gene ontology).^242^ Zhong et al. used transcriptomic and proteomic (DARTS) to discover that the natural product arctigenin played a role in renal protection through targeting PP2A.^243^ Previous research applied metabolomics and chemical proteomics (ABPP) to identify acetyl‐CoA carboxylase 1 (Acaca) and acetyl‐CoA carboxylase 2 (Acacb) as binding targets of PFOA^239^ (Table 2). Through these existing research reports, it can be shown that multi‐omics is a promising approach to the discovery of natural drug targets.

**TABLE 2:** Published reports on multi‐omics discovery of natural drug targets.

Methods	Compounds	Targets	Type of disease or model study	Potential applications	Ref
Transcriptomics and proteomics (DARTS)	Arctigenin	PP2A	Diabetic kidney disease	Hypertension; inflammation	^243^
Genomics and proteomics (DARTS)	Ecumicin	ClpC1	Tuberculosis	Lead compounds for antituberculosis drugs	^241^
Chemical proteomics and bioinformatics (gene ontology)	Baicalin	CPT1	Obesity and hepatic steatosis	Inflammation; antibacterial; obesity	^242^
Metabolomics and chemical proteomics (ABPP)	Perfluorooctanoic acid	Acaca, Acacb	PFOA‐induced liver toxicity	No	^239^
Proteomics (DARTS) and bioinformatics (gene docking)	Bithionol	NAD‐dependent dehydrogenases	Cryptococcus neoformans	Antifungal	^237^
ShRNA	Aurilide B	ATP1A1	Mitochondria‐mediated apoptosis	No	^244^
SiRNA	QS11	ARFGAP1	Breast cancer	No	^245^
CRISPR	Ispinesib	Kinesin‐5	Human cancer cells	Cancer	^246^
AI (machine learning)	β‐Lapachone	5‐lipoxygenase	Human cancer cells	Cancer	^247^
AI (deep learning)	Ziprasidone	5‐hydroxytryptamine receptor	No	No	^248^

## 6 CHALLENGES AND FUTURE DIRECTIONS OF MULTI‐OMICS

Although multi‐omics plays an important role in promoting research on human diseases, aging, discovery of natural drug targets, and so on, it also faces many problems and challenges. Here, we will discuss some challenges and future directions for multi‐omics research.

### 6.1 Challenges and opportunities for multi‐omics integration

**FIGURE 7:**
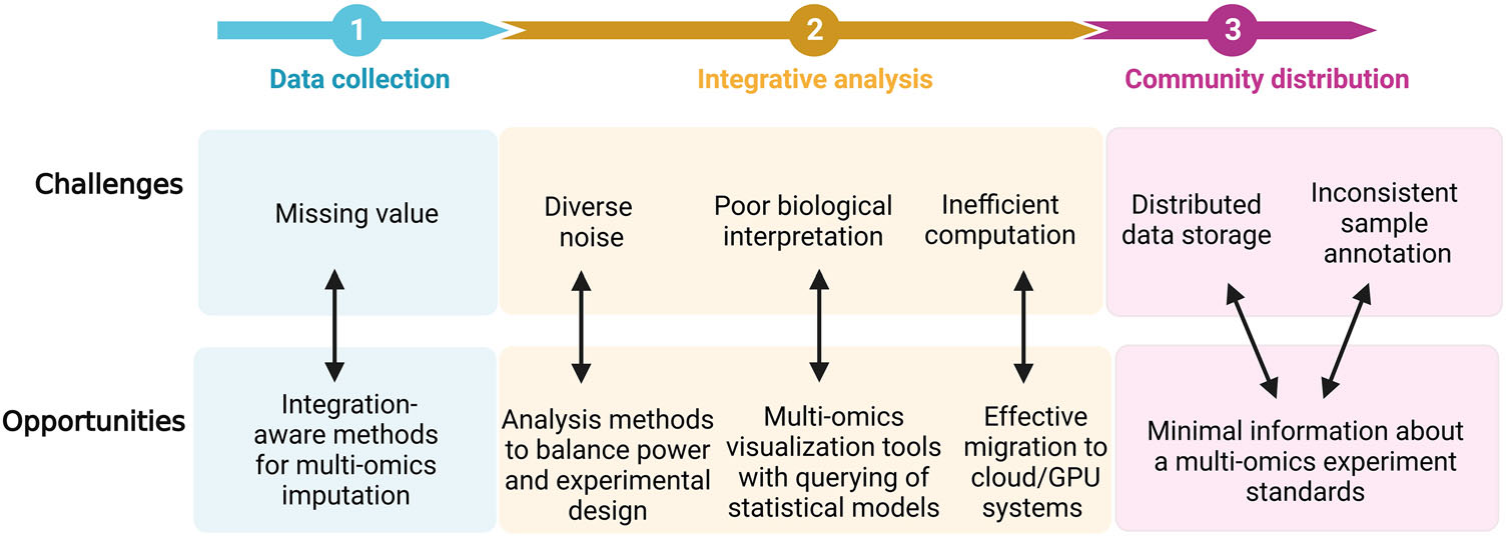
Challenges and opportunities for data integration of multi‐omics. The analysis challenges together with possible solutions are presented for three different levels, such as data collection, integrative analysis, and community distribution.

An early approach to multi‐omics analysis was the separate analysis of different types of data and then combined the results to obtain a comprehensive network of molecular interactions. With significant developments in this field, algorithmic meta‐analysis frameworks and methods have become the primary means of a comprehensive analysis of multi‐omics data.^92^, ^249^ However, multi‐omics integration analysis also faces some challenges and opportunities, such as missing value handling, heterogeneity between different omics, difficulties in interpreting multi‐omics models, and problems in data annotation, storage, and computing resources (Figure 7).^250^ In terms of missing values in omics data, the treatment of missing values is a more critical issue in the integrated analysis of multi‐omics data. Missing values can lead to undetectable features in some samples, for example, in proteomic and metabolomic analysis. The complete measurements are not available for some samples in multi‐omics, which may lead to more missing values. For example, in cohort studies, not all individuals can collect all types of omics data, so the number of individuals with complete records is usually much smaller than the overall sample size.^251^ And the missing values among various omics can influence the correlation analysis between different omics data, and interrupt the integration analysis between omics. While the missing values among omics can be solved by using a method of multiple imputation in a multiple factor analysis framework.^252^ Missing values in some samples may be due to quality control procedures, which usually can be solved by using the k‐nearest neighbor weighted imputation method for trans‐omics block missing data,^253^ such as MOFA, which can perform sample subgroup identification, data imputation, and abnormal sample detection for multi‐omics data.^135^ However, missing value imputation methods can reduce the reliability of the resulting data set,^254^ and generate data structures that violate the independence assumptions required by many statistical frameworks. Therefore, in integrated analyzes of multi‐omics data, sensitivity analysis was performed on missing value imputations to assess their impact on downstream analysis. In addition, for nonspecialist data processors, the researcher usually should try to avoid the intragroup or intergroup errors in samples from experimental manipulations, and minimize the testing times of omics instruments to avoid missing values due to batch benefits, especially for omics studies of large cohorts. In addition, the settings of instrument parameters need to be corrected in time to avoid data loss caused by instrument noise.

The heterogeneity among different omics is also an important factor affecting the integration of multi‐omics data. Different omics technologies have different precision levels, and the signal‐to‐noise ratio in multi‐omics measurements often affects the integration of multi‐omics data. For instance, proteomics favors the detection of abundantly expressed proteins, which is largely absent in transcriptomics. Due to this differential signature, analysis of the relationship between gene and protein expression is influenced by proteomic data.^81^ Algorithms have been developed to estimate the optimal sample size required for different omics to achieve a given statistical power.^81^ However, this treatment can lead to different amounts of sample data collected by different types of omics, resulting in many multi‐omics integration statistics methods not being applicable. Therefore, there is a need to find new analytical approaches that enable homogenous capabilities across multi‐omics data. In addition, the interpretability of multi‐omics models has outstanding advantages for understanding the complexity of life at the molecular level. In most cases, however, interpretability refers to the identification of biomarkers and the biological processes in which they are involved. Functional enrichment analysis is often used in the interpretation of multi‐omics data,^255^, ^256^ but these methods also have inherent limitations in providing mechanistic insights across molecular layers. Pathway diagrams have also been used for interpretation of multi‐omics data,^257^ but this approach lacks the flexibility to reveal novel regulatory relationships. In addition, Networks (such as Cytoscape^258^) that combine multi‐omics data, even though more general, can easily become too large, complex, and difficult to interpret. In the future, it is necessary to develop a calculation method combining the mathematical formula of multi‐layer adjustment with interactive visualization.

Data annotation and storage is also a challenge for multi‐omics data integration. Integrated annotation of multi‐omics data often requires software provided by technology suppliers; however, this software may be not publicly shared. Despite the abundance of publicly available multi‐omics studies and databases, retrieving multi‐omics data for specific biological entities remains a challenge. It is difficult to connect different types of omics data under the same research topic on various data platforms. Typically, various omics data sets are hosted in specific types of repositories, such as European Genome Archive (EGA, a sequencing database),^259^ MetaboLights (a metabolite database),^260^ and ProteomeXchang (a protein database).^261^ Therefore, hyperlinks are the only option to connect the same study across different omics databases. For example, construct metadata summaries to concatenate multiple omics data files from the same sample and then used them on a public database file.^262^ The European Bioinformatics Institute has developed a unified query system that can quickly find omics data sets responding to the same keyword across repositories.^263^ In the future, metadata description frameworks that define batch, experimental conditions, preprocessing, and replication will need to enable the linking of samples across omics data files and enable efficient reuse of multiple omics data.

In the future, with the expansion of samples and features, multi‐omics data will become more complex, and the development of precision medicine requires the integration of multi‐omics data sets from large cohorts of patients.^264^ The inclusion of other types of data, such as radiomics and single‐cell omics into multi‐omics research, will further increase the complexity of data processing. It is worth noting that cloud servers have been developed to help large‐scale data storage and data sharing, saving the operation and maintenance costs of computing infrastructure.^265^ Moreover, the processing of large amounts of data also requires high computing power, and cloud computing infrastructure needs to use sufficiently powerful computing units to achieve fast multi‐omics data processing, such as the often‐used graphics processing unit (GPU).^266^ GPUs can also meet the demand of the DL processing, which is often used in multi‐omics data analysis.^267^ In summary, developing efficient computing software and processing technology based on cloud services will facilitate the integration of multi‐omics data.

### 6.2 Single‐cell analysis as the future direction of multi‐omics

Single‐cell multi‐omics is the most promising development direction in the field of multi‐omics, which can infer regulatory models between multilayer molecules with better accuracy. Accounting for cell type heterogeneity will facilitate the modeling of complex biological processes (tumor^268^) and understanding of function in highly differentiated organs (brain^4^). Multiple omics studies on the same cell have been successful, such as combining RNA‐sequencing, DNA methylation, and/or protein abundance at the single‐cell level.^269^, ^270^ In view of the multiple omics data collected on the same cell, as they have the same cell information, the characteristics of different modalities can be matched,^271^ which is conducive to the subsequent integrated analysis, thereby improving the level of multi‐omics research. The main components of single‐cell multi‐omics analysis include single‐cell isolation, barcoding, and sequencing techniques for measuring multiple types of molecules from the same cell, as well as a comprehensive analysis of molecules measured at the single‐cell level to identify cell types and their functions related to pathophysiological processes based on molecular characteristics. The single‐cell omics usually focus on the data of mRNA‐genome, mRNA‐DNA methylation, mRNA‐chromatin accessibility, and mRNA‐protein. Here, we briefly introduce some detection technologies of the above single‐cell multi‐omics from the previous review^270^ (Table 3).

**TABLE 3:** Some technologies for single‐cell multi‐omics.

Category	Technologies	Separation methods	Feature	Ref
Genome‐transcriptome	G&T‐seq	Flow cytometry; bead‐based separation	Medium cell throughput; automation	^272^
	DR‐seq	Cell picking by pipette; preamplification and tagging of DNA and RNA followed by splitting	Low cell throughput	^273^
Transcriptome‐DNA methylome	scM&T‐seq	Flow cytometry; bead‐based separation followed by bisulfite treatment	Medium cell throughput; automation	^277^
	scMT‐seq	Micropipetting for isolation of single nuclei	Low cell throughput; partial automation	^281^
Transcriptome‐chromatin accessibility	sci‐CAR	Combinatorial indexing; lysate splitting followed by library preparation	High cell throughput	^282^
	SNARE‐seq	Microfluidic channels; open chromatin tagmentation followed by dual‐omics capture	High cell throughput	^283^
Transcriptome‐proteome	PEA/STA	Microfluidic channels; reverse transcription of PEA probe and RNA followed by targeted amplification	Medium cell throughput; automation	^279^
	PLAYR	Flow or mass cytometry; detection of amplified product of PLAYR probe pair and antibody staining	High cell throughput	^280^

For integrated analysis of single‐cell genome and transcriptome data, there have several approaches to achieve this analysis, such as genome and transcriptome sequencing (G&T‐seq),^272^ gDNA‐mRNA sequencing (DR‐seq),^273^ and so on. The approaches for single‐cell whole‐genome sequencing (scWGS) contain multiple displacement amplification,^274^ PicoPLEX (Rubicon Genomics PicoPLEX Kit), and so on, while the methods for scRNA‐seq include switching mechanism at the 5′end of the RNA transcript (Smart‐seq),^275^ and cell expression by linear amplification and sequencing (CEL‐seq).^276^ G&T‐seq uses the oligo‐dT‐coated magnetic beads to separate poly‐A‐tailed mRNAs from gDNA, and then sequencing by Smart‐seq2 and scWGS protocols, respectively. With this technology, Macaulay et al. reported that genomic imbalance on the chromosomes of a subpopulation of HCC38‐BL cells was consistent with changes in gene expression in unbalanced regions.^272^ The technology for integrating the signal‐cell data of transcriptome and epigenome is that the gDNA and RNA in the same single cell were separated and amplified using the G&T‐seq program, and then the single‐cell methylome and transcriptome sequencing (scBS‐seq) program was applied to the amplified gDNA to generate DNA methylome data.^277^ Using this technique, previous research found a link between epigenetic and transcriptional signatures associated with tissue‐specific mouse stem cell aging.^278^ The integration analysis of transcriptome and proteome data can be conducted by proximity extension assay/specific RNA target amplification (PEA/STA),^279^ proximity ligation assay for RNA (PLAYR),^280^ and so on. For PEA/STA method, adjacent‐dependent hybridization of DNA oligonucleotides attached to antibody pairs was performed using adjacent extension assay (PEA) labeled antibody pairs to convert proteins into DNA oligonucleotides, and RT mRNA using random RT primers to generate cDNA. Then, the DNA oligonucleotides and cDNA were amplified by PCR and quantified using quantitative PCR or sequencing. Using the PEA approach, Genshaft et al. found that a subpopulation of glioblastoma cells showed significant changes in mRNA and protein abundance following BMP4 treatment, and the proteins were able to define the response more accurately to BMP4.^279^

In addition, the analysis for single‐cell multi‐omics data is also complex. The general approaches can be divided as follows: correlation analysis between single‐cell mono‐omics data, combined analysis of all types of single‐cell omics data to generate an overall single‐cell atlas, analyze one type of single‐cell data and then integrate another single‐cell data type. For example, REAP‐seq was used to analyze PBMCs and calculated the Pearson correlation between single‐cell mRNA and protein expression of immune cell markers, and found that protein quantification was more sensitive than mRNA quantification for markers with low mRNA expression.^284^ Stoeckius et al. integrated cellular protein and transcriptome measurements into an efficient, single‐cell readout, and found that the protein expression level can be used to further subdivide the cell population identified by RNA‐seq with subtle mRNA expression differences, such as the (NK cell population.^285^ In addition, there are other methods for single‐cell multi‐omics integration, such as bi‐order canonical correlation analysis (bi‐CCA),^286^ UnpairReg (regression analysis on unpaired observations),^287^ PyLiger,^288^ nonnegative matrix factorization algorithm (UINMF),^289^ and so on.

However, although single‐cell multi‐omics data provide a wealth of biological information, their integrated analysis faces the same types of challenges described above. In addition, there is a lack of technology capable of generating multimodal data, resulting in comprehensive studies that often rely on the integration of modalities from different data sets. Notably, cross‐omics matching of cell types by using publicly available data from different modalities can greatly increase the number of single‐cell multi‐omics studies, such as MATCHER, an ensemble method dedicated to single‐cell‐specific data.^290^ Integration of single‐cell multi‐omics data also hinders the use of batch data processing methods due to specific technical limitations, such as cell‐level noise. Therefore, in the future, it also needs to develop relevant methods to interpret the biological significance of the data generated from a single type of cell.

In a word, the future field of single‐cell multi‐omics will not only address existing technical limitations, but also is a significant breakthrough in single‐cell ChIP‐sep, proteomics, metabolomics, and single‐cell omics combined with cellular‐level imaging and morphological analysis. Furthermore, future single‐cell multi‐omics will require the development of methods to decipher the biological interpretation of the complex signals linked between the different modalities. Finally, incorporating patient clinical data into single‐cell omics studies will help explain molecular regulatory models of health and disease at the cellular level.

### 6.3 Application of multi‐omics in precision medicine

The concept of precision medicine refers to an emerging approach to provide disease treatment options and prevention strategies based on individualized genetic, environmental, and lifestyle factors, in other words, the shift from one‐size‐fits‐all medicine to a broader paradigm of precision medicine (right drug, right patient, right dose, right time).^291^ Clinical data can be categorized into phenotypic (physiological assessment, disease scoring, imaging, health questionnaires, etc.) or multi‐omics molecular (genomics, transcriptomics, proteomics and metabolomics, etc.). Genetics research combined with clinical data provides the basis for precision medicine, such as translational genomics.^292^ And the study of blood proteomics can provide pathogenic target proteins for personalized therapy.^293^ Moreover, based on multi‐omics, there are significant advantages in explaining the molecular patterns of complex diseases, and identifying key nodes of disease at multiple levels of convergence, thus maximizing the precision of identifying new drug targets, endotypes, or biomarkers. However, the development of biomarkers based on multi‐omics is a key step in the realization of precision medicine.^294^ For example, the development of biomarker‐based companion diagnostics achieves precision medicine in the field of oncology.^295^, ^296^ While multi‐omics has difficulty in explaining the complex interactions between genetics, gene regulation, and proteins at multiple biological levels, networks can use their network topology to identify key nodes that are critical for screening drug targets and biomarkers for complex diseases.^297^, ^298^ In addition, based on the ML and DL methods discussed above, it is also helpful for multi‐omics to play an important role in the identification of biomarkers, and ultimately realize precision medicine.^129^ At present, there are also many review articles clarifying the wide application of multi‐omics in tumor and immune‐related precision medicine.^299^, ^300^ Similarly, in the field of neurodegenerative diseases, there are also research initiatives based on multi‐omics technology to realize precision medicine, such as the multi‐omics study of the gut microbial ecosystem in Parkinson's disease,^200^ brain tissue in Alzheimer's disease,^75^ induced pluripotent stem cell lines from ALS patients.^301^ In addition, multi‐omics has also been applied to precision medicine research on metabolic‐related diseases, such as type 2 diabetes.^302^

In general, multi‐omics plays a pivotal role in the development of precision medicine. With the development of high‐throughput technology and artificial intelligence algorithms, precision medicine based on multi‐omics will become the main trend of disease diagnosis, treatment, and prognosis in the future.

## 7 CONCLUSION

The study of biological multi‐omics data systematically reveals the physiological or pathological molecular map in health or disease state, and the development of multi‐omics technology is mainly to achieve precision medicine in the future. It is undeniable that multi‐omics is becoming increasingly important in medical research, and further development of its technology will likely explain the pathogenesis of major diseases (such as cancer and neurodegenerative diseases) and provide an important molecular theoretical basis for the clinical diagnosis, treatment, and prognosis. However, the current development of multi‐omics is also facing many challenges, such as the design of multi‐omics experiments mentioned above, especially the integrated analysis of multi‐omics data.

With the development of medical treatment and high‐throughput technology, various omics technologies are also integrated into disease research. In addition to the application of advanced omics technology, high‐level research reports often show their innovation and novelty in very sophisticated experimental design or analysis methods. Therefore, at the beginning of the experiment, special attention should be paid to whether the design of the experiment corresponds to the expected research purpose, after all, omics analysis is often both time‐consuming and costly. When conducting multi‐omics research, we must consider whether the type of omics, analysis techniques, and collected data indicators can achieve the set research goals or solve existing research problems. In other words, only a multi‐omics design approach suitable for explaining the purpose of the experiment is needed here, rather than just forcing the use of as many types of omics in the study as possible. It is believed that some suggestions on the design of multi‐omics experiments in this review may be helpful to guide researchers to carry out multi‐omics experiments in the future.

In addition, the large amount of data sets generated by multi‐omics also brings great difficulties to the data analysis. In this review, we also discussed some of the more commonly used multi‐omics data analysis and integration methods. For researchers with relatively weak computing algorithms, the way of multi‐omics data integration is often based on the correlation between various omics data and the discovery of related upper‐lower‐level regulatory networks, so as to further explain the biological significance of the data. However, researchers who are more proficient in bioinformatics and algorithms usually develop ML or DL‐related algorithms to integrate multi‐omics data, since ML and DL have significant advantages in multi‐omics data integration. For instance, ML and DL have excellent data processing capabilities for both linear and nonlinear data, and use supervised or unsupervised learning capabilities for disease prediction or biomarker discovery. Fortunately, open‐source platforms and software are also being developed to help noncomputational medical researchers integrate and analyze multi‐omics data, such as Hiplot and the “Wu Kong” platform (Table 4). Therefore, as more and more open‐source tools will be developed and accessible for the integrated analysis of multi‐omics data in the future, the application of multi‐omics will become more and more easy for most researchers.

**TABLE 4:** Open‐source tools for the integrated analysis of multi‐omics data.

Tool name	Omics type	Website	Advantages	Ref
OmicsAnalyst	Transcriptomics, proteomics, metabolomics, microbiome	https://www.omicsanalyst.ca	Correlation network analysis, cluster heat map analysis, and dimension reduction analysis all have comprehensive options for parameter customization, view customization, and target analysis.	^303^
OmicsNet 2.0	Genomics, transcriptomics, proteomics, metabolomics, microbiome	https://www.omicsnet.ca/	Web‐based multi‐omics analysis platform supporting 2D and 3D network visualization exploration. The Random Walk with Restart algorithm can be used to search for candidate disease markers.	^115^
Hiplot	Genomics, transcriptomics, proteomics	https://hiplot.com.cn/	Have a variety of omics analysis, bioinformation analysis modules, especially in the possession of high‐quality visual mapping tools.	^304^
Wu Kong	Genomics, transcriptomics, proteomics, phosphoproteomics Metabolomics	https://www.omicsolution.com/	Complete one‐click omics result generation, as well as a variety of statistical analysis, functional analysis, visualization analysis, clinical data analysis, multi‐omics cross‐association, and other modules.	^305^
OmicStudio	Genomics, transcriptomics, proteomics, metabolomics, microbiome	https://www.omicstudio.cn/	A variety of modules for single‐cell omics analysis, easy data entry types, and high‐quality visual mapping tools are available.	^306^
Majorbio Cloud	Genomics, transcriptomics, proteomics, metabolomics, microbiome	https://cloud.majorbio.com/	Provides interactive analysis reports that produce analysis results from bioinformation workflows into Web‐based interactive analysis reports.	^307^

In conclusion, with the development of high‐throughput technology, multi‐omics technology will become inseparable in the research of diseases, and finally realize precision medicine.

## AUTHOR CONTRIBUTIONS

C.C. and J.W. drafted the manuscript and Figures. D.P., X.W., Y.X., J.Y., and L.W. helped Figure modification. X.Y., M.Y., and G.P.L. revised the manuscript. All authors have read and approved the final manuscript.

## CONFLICT OF INTEREST STATEMENT

The authors declare no conflict of interest.

## ETHICS STATEMENT

Not appllicable.

## Data Availability

Data in this study are available upon reasonable request.
